# Efficacy of 1, 5, and 20 mg oral sildenafil in the treatment of adults with pulmonary arterial hypertension: a randomized, double-blind study with open-label extension

**DOI:** 10.1186/s12890-017-0374-x

**Published:** 2017-02-23

**Authors:** Carmine Dario Vizza, B. K. S. Sastry, Zeenat Safdar, Lutz Harnisch, Xiang Gao, Min Zhang, Manisha Lamba, Zhi-Cheng Jing

**Affiliations:** 1 Department of Cardiovascular and Respiratory Disease, University of Rome La Sapienza, Viale del Policlinico 155, 00161 Rome, Italy; 2 CARE Hospitals, Gandhi Bhavan Road Nampally, Hyderabad, India; 3 Baylor College of Medicine, 1 Baylor Plaza, Houston, TX 77030 USA; 4 Pfizer Ltd, Ramsgate Road, Sandwich Kent, CT13 9NJ UK; 5 Pfizer Inc, 558 Eastern Point Rd, Groton, CT 06340 USA; 6 Pfizer Inc, 10646 Science Center Dr, La Jolla Campus, San Diego, CA 92121 USA; 7 Shanghai Pulmonary Hospital, Tongji University School of Medicine, 507, Zhengmin Road, Shanghai, China

**Keywords:** Sildenafil, Clinical trial, Pulmonary hypertension, Exercise test, Echocardiography, Dose

## Abstract

**Background:**

In a previous study, 6-minute walk distance (6MWD) improvement with sildenafil was not dose dependent at the 3 doses tested (20, 40, and 80 mg 3 times daily [TID]). This study assessed whether lower doses were less effective than the approved 20-mg TID dosage.

**Methods:**

Treatment-naive patients with pulmonary arterial hypertension were randomized to 12 weeks of double-blind sildenafil 1, 5, or 20 mg TID; 12 weeks of open-label sildenafil 20 mg TID followed. Changes from baseline in 6-minute walk distance (6MWD) for sildenafil 1 or 5 mg versus 20 mg TID were compared using a Williams test. Hemodynamics, functional class, and biomarkers were assessed.

**Results:**

The study was prematurely terminated for non-safety reasons, with 129 of 219 planned patients treated. At week 12, 6MWD change from baseline was significantly greater for sildenafil 20 versus 1 mg (*P* = 0.011) but not versus 5 mg. At week 24, 6MWD increases from baseline were larger in those initially randomized to 20 versus 5 or 1 mg (74 vs 50 and 47 m, respectively). At week 12, changes in hemodynamic parameters were generally small and variable between treatment groups; odds ratios for improvement in functional class were not statistically significantly different. Improvements in B-type natriuretic peptide levels were significantly greater with sildenafil 20 versus 1 but not 5 mg.

**Conclusions:**

Sildenafil 20 mg TID appeared to be more effective than 1 mg TID for improving 6MWD; sildenafil 5 mg TID appeared to have similar clinical and hemodynamic effects as 20 mg TID.

**Trial registration:**

ClinicalTrials.gov NCT00430716 (Registration date: January 31, 2007).

**Electronic supplementary material:**

The online version of this article (doi:10.1186/s12890-017-0374-x) contains supplementary material, which is available to authorized users.

## Background

Pulmonary arterial hypertension (PAH) is a fatal disease in which increasing pulmonary vascular resistance ultimately culminates in right ventricular failure and death [1, 2]. The phosphodiesterase type 5 (PDE5) inhibitor sildenafil is approved to treat adult patients with PAH [2]; pediatric use is approved in the European Union.

In the 12-week, randomized, double-blind, SUPER-1 study [3], statistically significant improvements in 6-minute walk distance (6MWD) were observed with sildenafil versus placebo in treatment-naive patients at all 3 tested doses (20, 40, and 80 mg 3 times daily [TID]); improvements were similar among groups and did not appear to be dose related. However, hemodynamic parameters, including mean pulmonary arterial pressure (mPAP), cardiac index, and pulmonary vascular resistance index (PVRI), appeared to improve dose dependently with sildenafil treatment. Sildenafil 20 mg TID appeared to reach the plateau of the dose-response curve for 6MWD [3] and was confirmed by subsequent population pharmacokinetic and pharmacodynamic analysis [4].

This study was conducted to fulfill a postapproval commitment from the US Food and Drug Administration (FDA) to further explore the sildenafil dose-response curve. This multinational, randomized, double-blind study investigated whether low doses of sildenafil (1 and 5 mg TID) were less effective in adult patients with PAH than the currently approved 20-mg TID dose.

However, before completion of this low-dose study, results from another randomized, double-blind, placebo-controlled study (PACES-1) became available that supported approval of a clinical worsening indication by the FDA [5]. PACES-1 evaluated oral sildenafil in patients with PAH who were receiving stable epoprostenol therapy [6]. In PACES-1, ≥75% of patients were titrated from sildenafil 20 mg TID, received during the first 4 weeks, to sildenafil 40 mg TID at week 4, and then to sildenafil 80 mg TID at week 8 (and were maintained on this dose, as patients tolerated). After 16 weeks, 6MWD, hemodynamic parameters, and functional class improved. There was a significant delay in time to clinical worsening (TTCW) [6], defined as death, lung transplantation, hospitalization due to PAH, initiation of bosentan therapy, or clinical deterioration requiring a change in epoprostenol therapy, with sildenafil compared with placebo. The effect was apparent by week 4, when all patients were receiving sildenafil 20 mg TID (*P* = 0.0074) [4].

Following approval of the clinical worsening indication in the United States in 2009, the FDA released Pfizer from the postapproval commitment to conduct a low-dose study. The study was subsequently terminated (June 2010) based on the recommendation of the data monitoring committee (DMC) because sildenafil 20 mg TID had been shown to reduce time to clinical worsening in PACES-1 and also acknowledging that with recruitment issues the study was unlikely to meet original enrollment targets. Accumulated results are presented here.

## Methods

### Study design

**Fig. 1:**
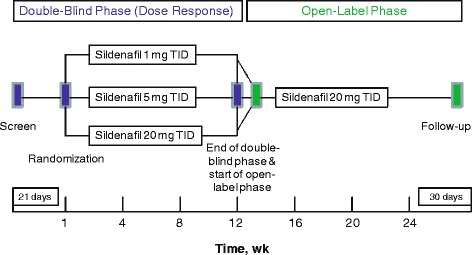
Study design. Legend: TID = 3 times daily

Patients were stratified by baseline 6MWD (<325 or ≥325 m) and PAH etiology and randomly assigned 1:1:1 to receive 12 weeks of treatment with sildenafil 1, 5, or 20 mg TID, respectively, during the double-blind phase of the study (Fig. 1). Patients who completed the double-blind phase were eligible for a 12-week, open-label extension in which they received sildenafil 20 mg TID. Patients who withdrew during the study were to be followed up for safety assessments 30 days after the last treatment date.

The primary objective of the study was to demonstrate a dose response for 6MWD for 1, 5, and 20 mg TID oral sildenafil. The hypothesis was that there is a dose that is significantly less effective than sildenafil 20 mg TID.

Secondary objectives included assessment of the safety and tolerability of low-dose sildenafil during the 12 weeks of treatment in patients with PAH and evaluation of the effects of sildenafil on perceived PAH-progression biomarkers (B-type natriuretic peptide [BNP]/pro-BNP levels and tricuspid annular plane systolic excursion [TAPSE]). The study protocol and amendments were reviewed and approved by the Institutional Review Board and/or Independent Ethics Committee at each participating center (Additional file 1); informed consent was obtained from all patients.

### Patients

Patients were aged >18 years with idiopathic or heritable PAH or PAH associated with connective tissue disease or surgical repair (≥5 years before enrollment) of atrial septal defect, ventricular septal defect, patent ductus arteriosus, or aorto-pulmonary window and 6MWD 100 to 450 m. PAH, defined as mPAP ≥25 mmHg and pulmonary artery wedge pressure ≤15 mmHg at rest (or a left ventricular end diastolic pressure <14 mmHg and absence of mitral stenosis on echocardiography), was confirmed by right heart catheterization (RHC) within 12 weeks before randomization. Patients had to be on stable (≥30 days before RHC) doses of background medication.

Patients were excluded for use of PAH-specific therapy, including prostacyclin, PDE5 inhibitors, and endothelin-receptor antagonists (ETRAs); nitrates or nitric oxide donors; protease inhibitors, such as ritonavir and saquinavir; ketoconazole, itraconazole, or other strong cytochrome P450 (CYP) 3A4 inhibitors; and alpha blockers. Patients previously receiving any of these drugs must have stopped use for ≥1 month before screening. Concomitant medications were to remain stable throughout the treatment phase of the study; patients withdrew if they required additional PAH-specific therapy.

### Assessments

Six-minute walk distance was assessed at baseline (day 1) and at weeks 4, 8, 12, 16, 20, and 24 as close to sildenafil trough levels as possible (ie, just before dosing and ≥4 h after the last scheduled dose). Borg dyspnea score was assessed at the end of the 6MWD evaluation. Hemodynamic status was assessed at baseline and week 12, using RHC. World Health Organization functional class was assessed at baseline; weeks 4, 8, 12, and 24; and follow-up.

Time to clinical worsening was assessed during the double-blind phase. Clinical worsening was defined as death, lung transplantation, hospitalization attributable to pulmonary hypertension, or initiation of prostacyclin or ETRA therapy.

Blood samples for determination of BNP/pro-BNP levels were collected at baseline and at weeks 1, 4, 8, 12, 16, 20, and 24. Echocardiography for TAPSE was performed at baseline and at weeks 4, 8, 12, and 24. A 2-dimensional Doppler examination was performed using an apical 4-chamber view. TAPSE index was measured as the total displacement of the tricuspid annulus (cm) from end diastole to end systole, with values representing the average TAPSE of 3 to 5 beats.

For pharmacokinetic analysis, blood samples were collected at the baseline visit (between 15 min and 3 h, >3 and 6 h, and >6 and 8 h postdose), week 1 (immediately after BNP/pro-BNP sampling), weeks 4 and 8 (immediately before 6MWD), and week 12 (between 15 min and 3 h and between >3 and 6 h postdose, immediately before 6MWD, between >6 and 8 h postdose, and during RHC assessment).

Adverse events (AEs) were monitored throughout the study. Laboratory testing and physical examinations were performed at screening, baseline, and weeks 4, 8, and 12.

### Dose selection

The relationship between 6MWD and sildenafil exposure could not be modeled because 6MWD had reached a plateau across all SUPER-1 dose groups [3]. Therefore, the relationship between PVRI and exposure was used to select doses predicting exposures from the population pharmacokinetic/pharmacodynamic model. The average sildenafil plasma concentration required to achieve 50% effect (EC_50_) on PVRI was approximately 3 ng/mL; at a 20-ng/mL concentration, sildenafil appeared to have a 90% maximal effect (EC_90_) on PVRI [4]. Therefore, after receipt of 20 mg TID, sildenafil concentrations were anticipated to be > EC_90_ for the entire 8-hour dosing interval; for 5 mg TID, above EC_50_ for the entire 8-hour dosing interval but < EC_90_ for most of the interval; and for 1 mg TID, sildenafil concentrations were anticipated to be at approximately EC_50_.

### Pharmacokinetic modeling

Population modeling characterized sildenafil pharmacokinetics; available sildenafil concentrations from all patients across all visits were merged to develop a nonlinear mixed effects model (NONMEM®, version 7.2; ICON Development Solutions, Ellicott City, MD). Estimation was performed for underlying pharmacokinetic parameters affecting the concentration-time profile. Only covariates that were previously reported to affect pharmacokinetic parameters [5] were tested in the model. To test for appropriateness, a visual predictive check was performed by calculating the median and 90% prediction interval from 500 simulations of the resulting population pharmacokinetic model.

### Statistical analysis

The estimated sample size was based on the primary endpoint and was determined using simulations. Assuming a treatment effect of 30 m for sildenafil 20 versus 1 mg TID, with a standard deviation of 60 m [3], 70 patients per group were required to detect a difference between treatments with 80% power at a 1-sided significance level of 2.5%. Allowing for 4% postrandomization nonevaluability, approximately 219 patients (73 per group) were required to be randomized.

For the primary endpoint, statistical significance was assessed with a 1-sided Williams trend test on the intent-to-treat (ITT) population; the ITT population consisted of randomized patients who received ≥1 dose of study medication. The highest noneffective dose (ie, the highest dose that is statistically significantly different from sildenafil 20 mg) was determined. Missing values were replaced according to the last observation carried forward (LOCF) in the primary analysis and via multiple imputation for sensitivity analyses.

Additionally, changes in the primary endpoint were modeled by analysis of covariance (ANCOVA) with randomized treatment, baseline 6MWD, and etiology as stratification factors. Pairwise treatment group differences were estimated. In the open-label phase, changes to week 24 (LOCF) were analyzed using this ANCOVA model (but also including week 12 [LOCF] in the model) if there was a nonmissing post–week-12 assessment.

Secondary endpoints (including hemodynamic parameters) were assessed in the ITT population using LOCF; covariates for each analysis included baseline value as well as the randomization strata of baseline 6MWD and etiology. Methods for LOCF, time to clinical worsening (TTCW), and Borg assessments are described in Additional file 2. For secondary endpoints, statistical significance was assessed based on nominal *P* values (<0.05; 2-sided) without adjustment for multiplicity.

## Results

The study was conducted at 34 centers in Europe, Asia, Russia, the United States, and Brazil. Of the planned 219 patients, 169 were screened, 130 were randomized, and 129 were treated (1 patient [sildenafil 1 mg] did not meet entry criteria). Treated patients were mostly female and mostly Asian; baseline cardiac index was significantly higher in the sildenafil 20-mg group versus the 1- and 5-mg groups (*P* = 0.0328 and 0.0030, respectively; Table 1).

**Table 1:** Baseline Patient Demographic and Clinical Characteristics

Baseline Characteristic	Sildenafil Dose, TID		
	1 mg (*n* = 41)	5 mg (*n* = 43)	20 mg (*n* = 45)
Women, n (%)	28 (68)	33 (77)	26 (58)
Age, y	42.5 (16.5)	44.4 (17.4)	46.4 (17.7)
Range	18–77	18–78	20–88
Race, n (%)			
White	11 (27)	11 (26)	14 (31)
Black	2 (5)	2 (5)	1 (2)
Asian	27 (66)	30 (70)	30 (67)
Other	1 (2)	0	0
Height, cm	159.0 (11.3)	160.2 (10.7)	160.7 (8.7)
Range	130.0–181.6	129.0–189.0	147.0–181.0
Weight, kg	61.7 (17.0)	63.1 (19.7)	61.4 (15.7)
Range	32.0–117.0	26.5–126.1	35.0–100.0
BMI, kg/m^2^	24.3 (5.6)	24.3 (6.6)	23.8 (6.2)
Range	15.6–35.8	13.3–42.1	15.6–38.6
WHO functional class, n (%)			
I	0	1 (2.3)	3 (6.7)
II	25 (61.0)	22 (51.2)	27 (60.0)
III	16 (39.0)	16 (37.2)	13 (28.9)
IV	0	1 (2.3)	0
Missing	0	3 (7.0)	2 (4.4)
Etiology, n (%)			
Idiopathic	30 (73)	31 (72)	34 (76)
Mean duration (range) since diagnosis, y	1.1 (0–6.7)	0.7 (0–6.5)	0.9 (0–14.9)
Associated with CTD	6 (15)	8 (19)	5 (11)
Mean duration (range) since diagnosis, y	0.6 (0–2.3)	0.4 (0–1.8)	0.4 (0–1.9)
Associated with surgical repair	5 (12)	4 (9)	6 (13)
Mean duration (range) since diagnosis, y	5.9 (0.3–14.2)	3.5 (0–7.3)	4.5 (0–15.7)
6MWD, m^a^	347.5 (67.3)	347.7 (73.4)	340.4 (76.3)
Range	167.5–441.5	109.0–455.0	114.0–429.0
Heart rate, bpm^b^	83.6 (17.2)	78.9 (16.4)	80.1 (15.0)
Range	48–122	42–113	53–110
RAP, mmHg^c^	10.5 (5.1)	10.1 (6.1)	8.4 (4.7)
Range	4.0–20.0	2.0–23.0	2.0–27.0
mPAP, mmHg^c^	57.2 (21.9)	55.4 (19.7)	51.1 (21.4)
Range	25.0–110.0	26.3–117.0	25.0–106.0
Cardiac index, L/min/m^2d^	2.1 (0.7)	2.3 (0.6)	2.8 (1.2)
Range	1.0–3.5	1.0–3.8	1.1–5.9
PVR, Wood units^e^	15.7 (9.9)	13.2 (8.3)	11.7 (9.1)
Range	3–43	3–48	2–35
MVO_2_, %^f^	63.4 (10.5)	63.0 (9.6)	64.3 (14.5)
Range	41–82	42–77	31–90
TAPSE index^g^	1.25 (0.62)	1.2 (0.71)	1.36 (0.83)
Range	0.1–2.6	0.1–2.5	0.2–2.8
Borg dyspnea score^h^	2.9 (2.5)	3.1 (1.9)	2.8 (2.1)
Range	0–10	0–8	0–9

All values are presented as mean (SD) unless stated otherwise

*6MWD* 6-minute walk distance, *BMI* body mass index, *bpm* beats per minute, *CTD* connective tissue disease, *mPAP* mean pulmonary arterial pressure, *MVO*_*2*_ mixed venous oxygen saturation, *PVR* pulmonary vascular resistance, *RAP* right atrial pressure, *TAPSE* tricuspid annular plane systolic excursion, *TID* 3 times daily, *WHO* World Health Organization

^a^ *n* = 2 and 3 patients missing a baseline assessment in sildenafil 5- and 20-mg groups, respectively

^b^ *n* = 33, 32, and 33 patients contributing data in sildenafil 1-, 5-, and 20-mg groups, respectively

^c^ *n* = 33, 33, and 34 patients contributing data in sildenafil 1-, 5-, and 20-mg groups, respectively

^d^ *n* = 33, 33, and 32 patients contributing data in sildenafil 1-, 5-, and 20-mg groups, respectively

^e^ *n* = 33, 32, and 32 patients contributing data in sildenafil 1-, 5-, and 20-mg groups, respectively

^f^ *n* = 33, 28, and 31 patients contributing data in sildenafil 1-, 5-, and 20-mg groups, respectively

^g^ *n* = 40 for all sildenafil groups

^h^ *n* = 41, 40, and 42 patients contributing data in sildenafil 1-, 5-, and 20-mg groups, respectively

**Fig. 2:**
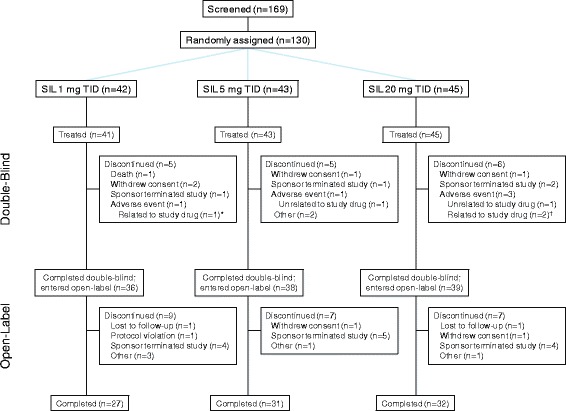
Patient disposition. Legend: TID = 3 times daily; SIL = sildenafil.*Right ventricular failure. ^†^Drug hypersensitivity (*n* = 1) and rash (*n* = 1)

Patient disposition is shown in Fig. 2. Two patients died during the double-blind phase (pneumonia [1 mg TID; death was the reason for discontinuation] and acute exacerbation of idiopathic pulmonary fibrosis [5 mg TID; patient was enrolled in error and received 4 days of treatment]), neither of which was considered to be treatment related; no deaths were reported in the open-label phase (Fig. 2).

### Sildenafil concentration

Overall, 129 patients provided 1068 sildenafil concentrations. A 1-compartment pharmacokinetic model adequately described the sparse data. From this model, the estimated apparent clearance was 43.9 (95% CI, 39.3–48.6) L/h, the apparent volume of distribution was 458 (95% CI, 393–523) L, and the absorption rate constant was 2.16 (95% CI, 1.48–2.84) h^−1^. Coadministration of weak or moderate CYP3A4 inhibitors (*n* = 12 patients/110 samples) reduced CL/F by 40.4% (95% CI, 19.2%–61.6%). The model supported dose proportionality of exposures.

The limit of quantification of the pharmacokinetic assay was 1 ng/mL; 134 samples were below the limit of quantification (BLQ). The majority of BLQ samples (approximately 75%) were measured at the 1-mg TID sildenafil dose, but had little effect on the population pharmacokinetic parameter estimates.

**Fig. 3:**
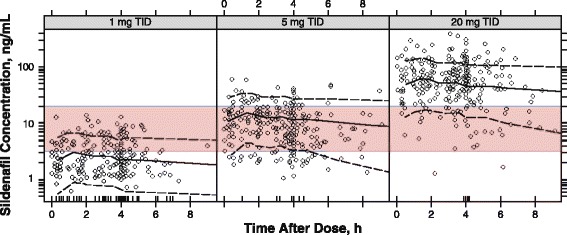
Plot of observed plasma sildenafil concentrations vs time after sildenafil dosing. Legend: Plasma sildenafil concentrations (*open circles*), sildenafil doses of 1 mg *(left*), 5 mg (*middle*), and 20 mg (*right*). Median (*solid line*) and 90% prediction intervals (*dashed lines*) from simulations are overlaid. Tick marks on the horizontal time axis indicate concentration measures below the limit of quantification. The shaded area shows the concentration range between 3 ng/mL and 20 ng/mL, which are the average sildenafil plasma concentrations required to achieve 50% effect (EC_50_) and 90% effect (EC90) on PVRI, respectively. TID = 3 times daily

Figure 3 represents the sildenafil concentration data. Because a small accumulation existed between the first (at baseline visit) and subsequent doses, only concentrations after the second and subsequent doses are shown (for data including baseline visit, see Additional file 3). Concentrations after concomitant administration of CYP3A4 inhibitors were adjusted for the estimated effect. Visual inspection of observed concentration distribution across each dose indicated consistency of the observed data with the model. In particular, in the 8 h after drug administration, most of the determinations in the 1-mg TID group had a concentration below 3 ng/mL, whereas in the 5-mg TID and 20-mg TID groups, most of the determinations had a concentration above 3 ng/mL, which is the average sildenafil plasma concentration required to achieve 50% effect (EC_50_) on PVRI [4]. An exploratory assessment (see Additional files 4 and 5) of the relationship between 6MWD, PVR, and steady-state concentrations revealed a significant relationship for 6MWD, whereas only a small trend could be seen for PVR across the concentration range observed (Additional files 6 and 7).

### Six-minute walk distance

**Fig. 4:**
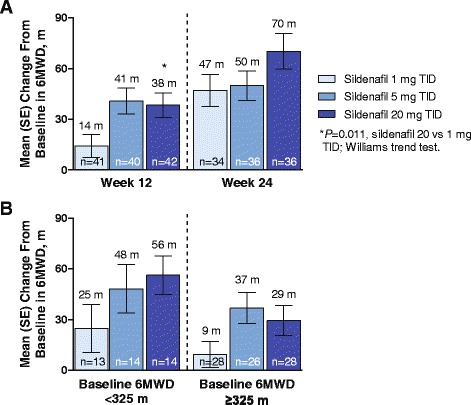
Mean change from baseline in 6MWD. Legend: Mean (SE) overall change from double-blind baseline in 6MWD in double-blind (week 12) and open-label (week 24) phases of the study (**a**), and change from baseline to week 12 in 6MWD by baseline 6MWD (**b**). All patients received sildenafil 20 mg TID in the open-label phase of the study (weeks 13–24). 6MWD = 6-minute walk distance; TID = 3 times daily

At week 12, compared with baseline, the increase in 6MWD was of a magnitude consistent with estimates of clinical significance [7, 8] in 5- and 20-mg TID groups and smaller although statistically significant in the 1-mg TID group. Among dose groups, the mean change in 6MWD from baseline was statistically significantly different only for the sildenafil 20- versus 1-mg group (Fig. 4a).

Analysis of change from baseline in 6MWD at week 12 showed a statistically significant (*P* = 0.011) difference between sildenafil 1 mg and 20 mg, but not sildenafil 5 mg and 20 mg (Table 2). The results were confirmed by an analysis of variance; the mean treatment difference between sildenafil 20 mg and 1 mg was 23 (3–43) m and between 20 mg and 5 mg was –3 (–23 to 17) m (*P* = 0.02 and 0.76, respectively).

**Table 2:** Change From Baseline^a^ in 6MWD at Week 12 (LOCF) Williams Trend Test

Value	Sildenafil Dose, TID		
	1 mg (*n* = 41)	5 mg (*n* = 43)	20 mg (*n* = 45)
Least squares mean	14.21	40.75	38.36
MLE mean^b^	14.21	39.52	39.52
Mean difference^c^	24.15	−1.17	—
Williams statistic	2.37	−0.11	—
97.5% lower confidence limit	3.37	−21.48	—
*P* value^d^	0.011	0.545	—

*6MWD* 6-minute walk distance, *LOCF* last observation carried forward, *MLE* maximum likelihood estimation, *TID* 3 times daily

^a^Baseline is the average of the screening and day 1 values

^b^MLE mean is defined as least squares mean if it satisfies descending response relationship for descending doses; if descending relationship does not hold, MLE mean is defined as weighted mean of adjacent least squares means

^c^Mean difference was calculated as the least squares mean for sildenafil 20 mg minus the MLE mean for sildenafil lower dose

^d^From directional test vs 20 mg TID

Patients with baseline 6MWD <325 m at baseline had greater increases in 6MWD after sildenafil treatment than patients with baseline 6MWD ≥325 m (Fig. 4b).

**Fig. 5:**
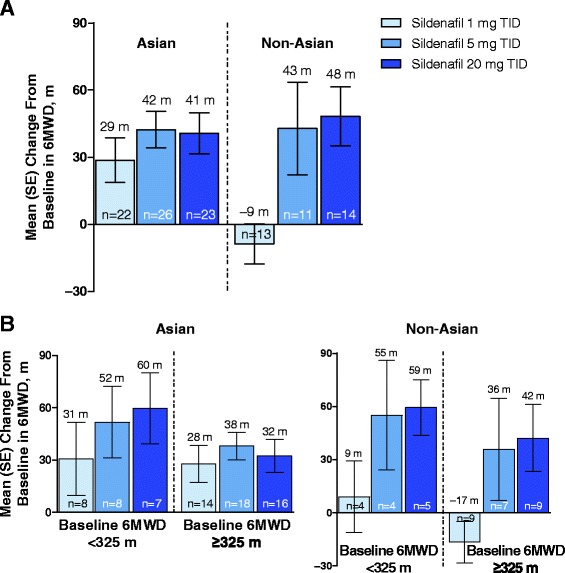
Mean change from baseline in 6MWD assessed by race. Legend: Mean (SE) overall change from double-blind baseline in 6MWD in the double-blind (week 12) phase of the study (**a**) and change from baseline to week 12 in 6MWD by baseline 6MWD (**b**) assessed by race (Asian vs non-Asian). 6MWD = 6-minute walk distance; TID = 3 times daily

Differences in 6MWD between Asian and non-Asian patients were noted for sildenafil 1 mg but not for 5 mg or 20 mg (Fig. 5a and b); the number of non-Asian patients was small.

During the open-label period (weeks 12 to 24), in which all patients received sildenafil 20 mg TID, patients who received sildenafil 1 mg TID during the double-blind phase (weeks 0 to 12) had a larger increase in 6MWD than patients who received sildenafil 5 mg TID (mean change, 31 vs 6 m, respectively); the magnitude of change was similar between patients who received sildenafil 1 mg and 20 mg TID in the double-blind phase (mean change, 31 vs 26 m; Fig. 4a).

### Secondary and tertiary evaluations

#### Hemodynamics

Compared with baseline, there was a trend toward reduction in pulmonary vascular resistance (PVR) at week 12 in all groups; the mean reduction was statistically significantly different from 0 only in the 20-mg TID group (ie, 95% CIs do not include 0). There were no statistically significant differences among treatment groups for change in PVR (Table 3). Changes at week 12 in the additional hemodynamic parameters were generally small and variable between groups.

**Table 3:** Adjusted Change From Baseline in Hemodynamic Parameters at Week 12

Baseline Characteristic	Sildenafil Dose, TID		
	1 mg	5 mg	20 mg
Heart rate			
n	33	32	33
LS mean (95% CI), bpm	3.4 (–1.1 to 7.9)	−0.7 (–5.2 to 3.7)	−5.0 (–9.3 to –0.8)
*P* value vs 20 mg TID	0.0019	0.1066	—
RAP			
n	33	33	34
LS mean (95% CI), mmHg	−0.5 (–2.3 to 1.2)	−0.8 (–2.5 to 0.9)	−1.7 (–3.3 to 0)
*P* value vs 20 mg TID	0.2741	0.4098	—
mPAP			
n	33	33	34
LS mean (95% CI), mmHg	−0.1 (–4.0 to 3.7)	−2.2 (–5.9 to 1.5)	−2.6 (–6.2 to 0.9)
*P* value vs 20 mg TID	0.2776	0.8458	—
Cardiac index			
n	32	31	30
LS mean (95% CI), L/min/m^2^	0.1 (–0.2 to 0.3)	0.1 (–0.1 to 0.4)	0.1 (–0.2 to 0.3)
*P* value vs 20 mg TID	0.9023	0.7590	—
PVR			
n	32	31	30
LS mean (95% CI), Wood units	−1.2 (–3.3 to 0.9)	−2.0 (–4.1 to 0)	−2.4 (–4.3 to –0.4)
*P* value vs 20 mg TID	0.3694	0.8010	—
PVRI			
n	32	31	30
LS mean (95% CI), Wood units*m^2^	−1.7 (–4.9 to 1.5)	−3.1 (–6.2 to 0)	−3.5 (–6.4 to –0.5)
*P* value vs 20 mg TID	0.3868	0.8628	—
MVO_2_			
n	33	28	31
LS mean (95% CI), %	1.5 (–2.2 to 5.2)	3.0 (–0.8 to 6.7)	3.0 (–0.4 to 6.4)
*P* value vs 20 mg TID	0.4918	0.9791	—

*bpm* beats per minute, *LS* least squares, *mPAP* mean pulmonary arterial pressure, *MVO*_*2*_ mixed venous oxygen saturation, *PVR* pulmonary vascular resistance, *PVRI* PVR index, *RAP* right atrial pressure, *TID* 3 times daily

#### Functional class and clinical worsening

Most patients in each treatment group remained in the same functional class from baseline to week 12; the same was true through week 24 (Table 4). Odds ratios (ORs) showed no significant differences for functional class between sildenafil 20 mg and the 5-mg (OR, 1.08 [95% CI, 0.35–3.32]; *P* = 0.897) or 1-mg (OR, 1.55 [95% CI, 0.50–7.78]; *P* = 0.448) dose at week 12. Similarly, there were no differences between sildenafil 20 mg and the 5-mg (OR, 1.31 [95% CI, 0.42–4.05]; *P* = 0.639) or 1-mg (OR, 0.93 [95% CI, 0.30–2.91]; *P* = 0.899) dose at week 24. Four patients (sildenafil 1 mg and 5 mg, *n* = 1 each; sildenafil 20 mg, *n* = 2) reported events defined as clinical worsening (initiation of ETRA therapy [sildenafil 5-mg patient] and hospitalization due to PAH [all others]).

**Table 4:** Change From Baseline to Weeks 12 and 24 in Functional Class (LOCF)

Change, n (%)	Sildenafil Dose, TID					
	Double-Blind Phase (Week 12)			Open-Label Phase (Week 24)		
	1 mg (*n* = 41)	5 mg (*n* = 43)	20 mg (*n* = 45)	1 mg (*n* = 41)	5 mg (*n* = 43)	20 mg (*n* = 45)
Worsened 2 classes	0	0	0	0	0	1 (2)
Worsened 1 class	1 (2)	3 (7)	2 (4)	0	3 (7)	1 (2)
No change	35 (85)	27 (63)	35 (78)	23 (56)	19 (44)	22 (49)
Improved 1 class	4 (10)	10 (23)	6 (13)	11 (27)	13 (30)	11 (24)
Improved 2 classes	1 (2)	0	0	1 (2)	1 (2)	1 (2)
Missing	0	3 (7)	2 (4)	6 (15)	7 (16)	9 (20)

*LOCF* last observation carried forward, *TID* 3 times daily

#### Neurohormones

**Fig. 6:**
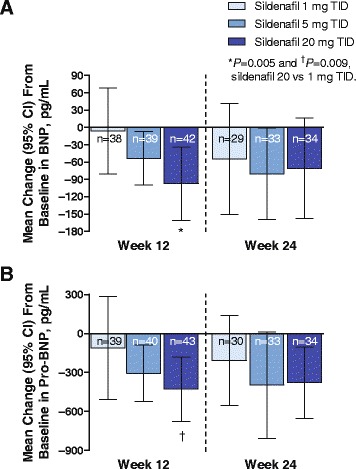
Changes from baseline in BNP (**a**) and pro-BNP (**b**) during double-blind (week 12) and open-label (week 24) phases of the study. All patients received sildenafil 20 mg TID in the open-label phase of the study (weeks 13–24). BNP = B-type natriuretic peptide; TID = 3 times daily

Decreases from baseline in BNP occurred in all groups at week 12; the response was dose related (Fig. 6a). The sildenafil 20-mg group was statistically significantly (*P* = 0.005) different from the 1-mg but not the 5-mg group (*P* = 0.496). At week 24, changes from baseline for sildenafil 20 mg were not significantly different among groups.

Pro-BNP decreases occurred in all groups at week 12 and were dose related (Fig. 6b). Differences were significant when sildenafil 20 mg was compared with 1 but not 5 mg (*P* = 0.009 and 0.414, respectively). At week 24, changes from baseline were not significantly different among groups.

#### Echocardiography

There was a trend toward a mean increase in TAPSE in all groups, but there were no statistically significant differences in mean TAPSE index among groups (mean [95% CI] increases of 0.14 [0.02–0.26], 0.17 [0.06–0.28], and 0.04 [–0.08 to 0.16] cm for sildenafil 1, 5, and 20 mg TID, respectively, at week 12 [LOCF] and 0.21 [0.06–0.37], 0.40 [0.19–0.61], and 0.15 [–0.09 to 0.39] at week 24 [LOCF]).

#### Borg dyspnea score

Borg dyspnea scores trended toward reduction in all groups (mean [95% CI] changes of –0.28 [–0.76 to 0.20], –0.89 [–1.35 to –0.43], and –0.43 [–0.94 to 0.08] for sildenafil 1, 5, and 20 mg TID, respectively, at week 12 [LOCF] and –1.10 [–1.75 to –0.46], –1.07 [–1.55 to –0.58], and –0.28 [–0.75 to 0.20] at week 24 [LOCF]), with no significant differences between sildenafil 1- and 5-mg TID groups compared with sildenafil 20 mg TID.

### Correlations among parameters

Baseline 6MWD was weakly correlated with BNP (*r* = –0.19; *P* = 0.0393) and pro-BNP (*r* = –0.22; *P* = 0.0145). The change in 6MWD at week 12 was also weakly correlated with changes at week 12 in BNP (*r* = –0.18; *P* = 0.0499) and pro-BNP (*r* = –0.22; *P* = 0.0193).

### Adverse events

The overall number of AEs and numbers of patients reporting AEs were similar between treatment groups in the double-blind and open-label portions of the study; treatment-related AEs (number of AEs and patients reporting AEs) increased with increasing dose (Table 5). Sildenafil was generally well tolerated, with most AEs being mild or moderate in severity. Dyspnea was the most common AE reported in both phases of the study; headache was the most common treatment-related AE (Table 5). No patients discontinued as a result of abnormal laboratory test results, and there was no evidence of dose-related increase in laboratory test abnormalities with increasing sildenafil dose.

**Table 5:** Adverse Event Summary

All-Cause (Treatment-Related) AEs, n	Sildenafil Dose, TID					
	Double-Blind Phase (Week 12)			Open-Label Phase^a^ (Week 24)		
	1 mg (*n* = 41)	5 mg (*n* = 43)	20 mg (*n* = 45)	1 mg (*n* = 41)	5 mg (*n* = 43)	20 mg (*n* = 45)
Patients with AEs	17 (9)	17 (10)	19 (14)	23 (11)	22 (12)	22 (15)
Patients with serious AEs	4 (0)	2 (0)	3 (1)	6 (0)	3 (0)	5 (2)
Discontinuations due to AEs	1 (1)	1 (0)	3 (2)	1 (1)	1 (0)	3 (2)
Deaths	1 (0)	1 (0)	0	0	0	0
Number of AEs	46 (12)	41 (17)	47 (24)	90 (19)	69 (27)	74 (31)
AEs occurring in ≥3 patients						
Anemia	1 (0)	0 (0)	3 (1)	1 (0)	1 (0)	3 (1)
Fatigue	2 (1)	1 (0)	0 (0)	3 (1)	1 (0)	0 (0)
Nasopharyngitis	2 (0)	1 (0)	1 (0)	2 (0)	1 (0)	3 (0)
Dizziness	2 (1)	1 (0)	1 (1)	3 (1)	2 (1)	2 (2)
Dyspnea	2 (0)	3 (0)	3 (0)	2 (0)	4 (1)	3 (0)
Headache	1 (1)	1 (1)	3 (3)	2 (2)	3 (2)	3 (3)
Epistaxis	0 (0)	2 (2)	0 (0)	1 (0)	3 (3)	0 (0)
Back pain	0 (0)	1 (0)	2 (1)	1 (0)	1 (0)	3 (2)

*AE* adverse event, *TID* 3 times daily

^a^Includes AEs from the double-blind and open-label portions of the study

## Discussion

Sildenafil is one of the most widely used drugs in the treatment of PAH. The dose of 20 mg TID was approved based on the results of the SUPER-1 study which demonstrated that Sildenafil 20 mg TID appeared to reach the plateau of the dose-response curve for 6MWD, despite the larger hemodynamic effects seen with the highest dosage (80 mg TID). These results raise the question as to whether a lower dosage could have a similar effect on 6MWD compared to the approved dose. This aspect was addressed in the present study.

We found a significant increase from baseline in 6MWD at 12 weeks with all sildenafil doses; however, only at higher doses (5 and 20 mg TID) was the improvement of a magnitude considered to be clinically relevant (~40 m) [7, 8]. In the absence of a placebo control arm, the small non-clinically significant increase in 6MWD in the 1 mg TID group in the double blind phase should be interpreted with caution as being a treatment effect as it is possible that this improvement could be seen as a “placebo effect” due to participation in an RCT.

Among dose groups, the change in 6MWD from baseline was significant only with sildenafil 20 mg TID compared with sildenafil 1 mg TID. A Williams trend test confirmed that sildenafil 1 mg TID was the only dose statistically inferior to the approved dose of 20 mg TID. Generally, patients had greater improvements in hemodynamic parameters with sildenafil 20 mg TID versus 1 mg TID; however, these improvements were not statistically significantly different. Significant differences were observed between sildenafil 1 mg TID and 20 mg TID for neurohormones at week 12.

There were no statistically significant differences between sildenafil 20 and 5 mg TID in 6MWD, hemodynamics, or changes in functional class.

Results from pharmacokinetic modeling showed that the observed exposure with sildenafil 1 mg TID was slightly below EC_50_ for maximal PVR change, the observed exposure with sildenafil 5 mg TID was above EC_50_ and approaching EC_90_, and the observed exposure with sildenafil 20 mg TID was mainly above EC_90_.

The pharmacokinetic data justify the different clinical responses between sildenafil 1 and 20 mg TID and explain the small difference observed between sildenafil 20 and 5 mg TID because most of the patients on 5 mg TID had a sildenafil plasma level between 3 and 20 ng/mL.

A significant correlation among mean sildenafil plasma concentration and 6MWD could be observed, although the relationship between average sildenafil plasma concentration and PVR appeared to show only a shallow trend. Whether this was due to the missing placebo group or was a consequence of the smaller sample size and larger variability on PVR cannot be concluded but should be interpreted on the basis of the complex interplay between pharmacokinetics and pharmacodynamics. The vasodilator effect is the result of the interplay of several factors: tissue penetration of the drug, density and activity of PDE5 enzyme, and severity of vascular lesions.

Smaller improvement in 6MWD at week 12 with sildenafil 20 mg TID was observed in this study (38 m) compared with SUPER-1 (45 m); however, patient populations differed. Both studies had similar baseline 6MWD, but a greater proportion of patients in this study had baseline functional class II status compared with those in SUPER-1 (57% vs 39%, respectively); therefore, patients in this study had lower-than-expected 6MWD at baseline. Patients in our study were also younger (45 vs 49 years), with a shorter time since diagnosis (median, 0.17 vs 0.85 years) and an increased percentage of Asian patients (67% vs 7%). Geographic variation in 6MWD has been described for patients with PAH and was reported to be independent of anthropometric factors [9]. Although few non-Asian patients enrolled in this study, 6MWD did not appear to differ between groups, with the exception of sildenafil 1 mg TID (Fig. 5).

Interestingly, results from the open-label phase suggest the possibility of further improvement in 6MWD after the first 3 months of therapy with sildenafil 20 mg TID. The mean increase in 6MWD from baseline at the end of the double-blind phase (41 m) was maintained in the sildenafil 5-mg group uptitrated to sildenafil 20 mg TID in the extension study (50 m), yet larger increases were observed from the end of the double-blind study to the end of the open-label study in the sildenafil 1- and 20-mg groups (from 14–47 m and from 38–70 m, respectively). Thus, sildenafil 20 mg TID maintains treatment effects regardless of prior low-dose treatment. However, 6MWD did not increase to the same degree in patients previously treated with lower doses as in patients who continuously received 20 mg TID, suggesting that a longer duration of an adequate dose may confer a larger improvement in 6MWD. Interestingly, the total improvement observed after 24 weeks in the 20-mg group (70 m) was larger than in the SUPER-1 study at 12 weeks (48 m) or 1 year (51 m) for all sildenafil doses combined. It may be possible that in a population of young and mainly incident cases, as in our study, further improvements in 6MWD may be observed with continued sildenafil treatment.

Decreases for BNP and pro-BNP versus baseline were significantly higher with sildenafil 20 mg versus 1 mg TID at week 12, paralleling findings with 6MWD. BNP levels similarly paralleled improvements (BNP levels decreased) or worsening (BNP levels increased) in pulmonary hemodynamics and functional parameters, including 6MWD, in patients with PAH in a previous study [10]. Elevated plasma BNP levels are associated with increased mortality in patients with PAH, and a decrease in BNP levels after therapy is associated with improved survival [11, 12]. Pro-BNP levels have recently been shown to identify poor outcome in patients with PAH [13, 14]. Longer-term follow-up of patients from our study is not ongoing, which prevents any correlation with mortality.

The main limitation of the present study is its premature termination. The study was designed to assess the relative efficacy of sildenafil 20 mg TID and lower doses and powered for the primary endpoint but the sample size was not reached because of premature termination [4, 6]. Looking at the results, this does not seem a major issue, as the difference in the primary and secondary endpoints between 1 mgTID and 20 mg TID is statistically significant and coherent. Regarding the comparison between the 5-mg and 20-mg groups, the differences were small enough that, even with the completion of the study, similar results may have been observed. A noninferiority study comparing sildenafil 5 mg TID versus 20 mg TID would require an unrealistically large sample size for a rare disease like PAH. Estimating from the results of the current study, 382 patients would be required for a study with a noninferiority margin of 15 m at 90% power and a 1-sided significance level of 0.05, assuming a true difference (5 vs 20 mg TID) of 0 m and a standard deviation of 50 m. The required sample size would increase if patient dropout was considered or if a smaller noninferiority margin was desired.

## Conclusion

Despite this study having the limitation of premature termination, sildenafil 1 mg TID, but not 5 mg TID, was shown to be inferior to 20 mg TID for improvement in 6MWD in patients with PAH. Sildenafil 5 mg TID appeared to have similar clinical and hemodynamic effects as 20 mg TID. Interestingly, 6MWD results from the open-label phase of the study suggest that patients on the approved sildenafil dose (20 mg TID) continued to show clinical improvement after the first 12 weeks of treatment. Hence, the question remains whether doses lower than 20 mg TID have therapeutic value and needs to be seen in light of the current therapeutic approach in PAH.

## Additional files

Additional file 1: List of Investigators and Corresponding Ethics Committees or Institutional Review Boards. (PDF 140 kb)
Additional file 2: Supplemental Methods. Methods for LOCF, time to clinical worsening (TTCW), and Borg assessments. (DOCX 14 kb)
Additional file 3: Figure S1. Relationship of change from baseline in 6MWD and sildenafil average steady-state concentration. (DOCX 121 kb)
Additional file 4: Figure S2. Relationship of change from baseline in PVR and sildenafil average steady-state concentration. (DOCX 125 kb)
Additional file 5: Figure S3. Plot of observed plasma sildenafil concentrations (open circles) vs time after sildenafil doses of 1 mg TID (bottom panels), 5 mg TID (middle panels), and 20 mg TID (top panels). (DOCX 179 kb)
Additional file 6: Table S1. Results of a linear model, regressing 6MWD change from baseline against sildenafil average steady-state concentrations. (DOCX 14 kb)
Additional file 7: Table S2. Results of a linear model, regressing PVR change from baseline against sildenafil average steady state concentrations. (DOCX 14 kb)

## ᅟ

6MWD: 6-minute walk distance
AE: Adverse event
ANCOVA: Analysis of covariance
BLQ: Below the limit of quantification
BNP: B-type natriuretic peptide
CYP: Cytochrome P450
DMC: Data monitoring committee
ETRA: Endothelin-receptor antagonists
FDA: US Food and Drug Administration
ITT: Intent to treat
LOCF: Last observation carried forward
mPAP: Mean pulmonary arterial pressure
OR: Odds ratio
PAH: Pulmonary arterial hypertension
PDE5: Phosphodiesterase type 5
PVR: Pulmonary vascular resistance
PVRI: Pulmonary vascular resistance index
RHC: Right heart catheterization
TAPSE: Tricuspid annular plane systolic excursion
TID: 3 times daily
TTCW: Time to clinical worsening
