# Supplementary material for: Efficacy of 1, 5, and 20 mg oral sildenafil in the treatment of adults with pulmonary arterial hypertension: a randomized, double-blind study with open-label extension
Source: BMC Pulm Med. 2017 Feb 23;17:44. doi: 10.1186/s12890-017-0374-x (PMC5322647; doi:10.1186/s12890-017-0374-x)
Supplement: Additional file 1: — List of Investigators and Corresponding Ethics Committees or Institutional Review Boards. (PDF 140 kb) [file 12890_2017_374_MOESM1_ESM.pdf]

090177e181b842ab\Approved\Approved On: 17-Mar-2011 06:18

A4 LIST OF INVESTIGATORS AND CORRESPONDING ETHICS COMMITTEES OR INSTITUTIONAL REVIEW BOARDS

Belgium

Coordinating Investigators:
<None Entered>

Center	Principal Investigator	Co-Investigator(s)	Sub-Investigator(s)	Address(es)	Institutional Review Board or Ethics Committee Address(es)
1033 *	Prof. Marion Delcroix			Universitaire Ziekenhuizen<br>Leuven - Campus Gasthuisberg<br>Pneumologie<br>Herestraat 49<br>Leuven, 3000<br>BELGIUM	Commissie Medische Ethiek van de<br>Universitaire Ziekenhuizen<br>KULeuven<br>U.Z. Gasthuisberg E330<br>Herestraat 49<br>Leuven, 3000<br>BELGIUM<br><br>Universitaire Ziekenhuizen Leuven -<br>Campus Gasthuisberg<br>Commissie voor Medische Ethiek /<br>Klinisch Onderzoek<br>Herestraat 49<br>Leuven, 3000<br>BELGIUM
1034 *	Prof. Robert Naeije		Sandrine Huez<br>Dr. Jean-luc Vachiery	Hopital Erasme / Service de<br>Cardiologie<br>Route de Lennik, 808<br>Bruxelles, 1070<br>BELGIUM	Universitaire Ziekenhuizen Leuven -<br>Campus Gasthuisberg<br>Commissie voor Medische Ethiek /<br>Klinisch Onderzoek<br>Herestraat 49<br>Leuven, 3000<br>BELGIUM

Brazil

Coordinating Investigators:
<None Entered>

Center	Principal Investigator	Co-Investigator(s)	Sub-Investigator(s)	Address(es)	Institutional Review Board or Ethics Committee Address(es)
1028	Dr. Maria Virginia Tavares Santana		Dr. Maria E. M. Albrecht<br>Dr. Thais T. Costa<br>Dr. Almir S. Ferraz<br>Luis C P Lima<br>Dr. Ricardo F. Martins<br>Dr. Romeu S. Meneghelo<br>Dr. Marly A. Miaira<br>Fernando A Moreira<br>Dr. Carlos Augusto Cardoso Pedra<br>Dr. Sérgio C. Pontes Júnior	Instituto Dante Pazzanese de Cardiologia<br>Av Doutor Dante Pazzanese, 500 - Predio I<br>Vila Mariana<br>Sao Paulo, SP 04012-909<br>BRAZIL	Comite de Etica em Pesquisa do Instituto Dante Pazzanese de Cardiologia<br>Av Doutor Dante Pazzanese, 500<br>6º andar - Torre - Ibirapuera<br>Sao Paulo, SP 04012-180<br>BRAZIL
1029	Jose Miguel Chatkin		Dr. Daniela Cavalet Blanco<br>Dr Paulo Ricardo Avancini Caramori<br>Dr. Gustavo Chatkin<br>Leandro Genehr Fritscher<br>Fabio Maraschin Haggstram<br>Dr Flavio Jose Petersen Velho	Hospital São Lucas da PUCRS<br>Av. Ipiranga, 6690 - 4 andar<br>Bairro Azenha<br>Porto Alegre, RS 90610-000<br>BRAZIL	Comite de Etica em Pesquisa em Seres Humanos da PUCRS<br>Av: Ipiranga, 6690 - 3º andar<br>Bairro Azenha<br>Porto Alegre, RS 90610-000<br>BRAZIL
1091 *	Dr. Frederico T. A. F. Campos		Virginia Pacheco Guimarães<br>Eliane Viana Mancuzo<br>Edmundo C. Oliveira	Hospital Madre Teresa<br>Departamento de Pneumologia<br>Av. Raja Gabaglia, 1002<br>Bairro Gutierrez<br>Belo Horizonte, MG 30380-090<br>BRAZIL	Comitê de Ética em Pesquisa do Hospital Madre Teresa<br>Av. Raja Gabaglia, 1002<br>Bairro Gutierrez<br>Belo Horizonte, MG 30380-090<br>BRAZIL

* Did not randomize subjects

090177e181b842ab\Approved\Approved On: 17-Mar-2011 06:18

090177e181b842ab\Approved\Approved On: 17-Mar-2011 06:18

Bulgaria

Coordinating Investigators:
<None Entered>

Center	Principal Investigator	Co-Investigator(s)	Sub-Investigator(s)	Address(es)	Institutional Review Board or Ethics Committee Address(es)
1069 *	D-r Georgi Grigorov		Todor Draganov<br>Dr. Mima Njagina<br>Dr. Georgi Todorov	Vtora mnogoprofilna bolnitsa za aktivno lechenie, Klinika po kardiologia<br>bul. "Hristo Botev" ¿120<br>Sofia, 1202<br>BULGARIA	2nd Multifunctional Hospital for Active Treatment, Sofia<br>Ethics Committee<br>120 Hristo Botev Blvd.<br>Sofia, 1202<br>BULGARIA<br><br>Ethics Committee for Multicenter Trials<br>ul. "Damyan Gruev" 8<br>Sofia, 1303<br>BULGARIA
1070 *	Dr. Sotir Todorov Marchev		Dr. Bojidar Ivanov Dimov<br>Ventsislav Mirchev<br>Vera Rangelova	Peta mnogoprofilna bolnitsa za aktivno lechenie, Klinika po kardiologia<br>bul. General Stoletov 67A<br>Sofia, 1233<br>BULGARIA	5th Multifunctional Hospital for Active Treatment, Sofia<br>Ethics Committee<br>67A Stoletov Blvd.<br>Sofia, 1233<br>BULGARIA<br><br>Ethics Committee for Multicenter Trials<br>ul. "Damyan Gruev" 8<br>Sofia, 1303<br>BULGARIA

Center	Principal Investigator	Co-Investigator(s)	Sub-Investigator(s)	Address(es)	Institutional Review Board or Ethics Committee Address(es)
1073 *	Dr. Zlatka Iankova		Dr. Milena Dimitrova<br>Anna Kostova Kostova<br>Dr. Neli Boyanova Manolova<br>Dots. Stoyan Milanov<br>Dots. Maria Hristova Milanova<br>Diana Slaveva Mladenova MD<br>Dimitar Tsachev MD	Mnogoprofilna bolnitsa za aktivno lechenie i speshna meditsina "N.I.Pirogov" Klinika po speshni vatreshni bolesti, Otdelenie po pulmologia<br>bul. General Totoleben 21<br>Sofia, 1606<br>BULGARIA	Ethics Committee at Mnogoprofilna bolnitsa za aktivno lechenie i speshna meditsina "N.I.Pirogov" Mnogoprofilna bolnitsa za aktivno lechenie i speshna meditsina "N.I.Pirogov"<br>bul. General Totleben 21<br>Sofia, 1606<br>BULGARIA<br><br>Ethics Committee for Multicenter Trials<br>ul. "Damyan Gruev" 8<br>Sofia, 1303<br>BULGARIA
1076 *	Dr. Borislav Georgiev		Elena Kostova	Spetsializirana bolnitsa za aktivno lechenie na sardechno-sadovi zabolyavania Klinika po kardiologia<br>ul. Konyovitsa 65<br>Sofia, 1309<br>BULGARIA	Ethics Committee at Specialized Hospital for Active Tteatment of Cardio-Vascular Diseases<br>65 Koniovitza Street<br>Sofia, 1309<br>BULGARIA<br><br>Ethics Committee for Multicenter Trials<br>ul. "Damyan Gruev" 8<br>Sofia, 1303<br>BULGARIA

090177e181b842ab\Approved\Approved On: 17-Mar-2011 06:18

China

Coordinating Investigators:
Lizhi Dai
Xin Jiang

Center	Principal Investigator	Co-Investigator(s)	Sub-Investigator(s)	Address(es)	Institutional Review Board or Ethics Committee Address(es)
1101	Prof. Zhicheng Jing		Dong Liu<br>Xiqi Xu<br>Qinhua Zhao	Shanghai Pulmonology Hospital<br>Department of Pulmonary Circulation<br>No.507 Zhengmin Road Yangpu District<br>Shanghai, 200433<br>CHINA	Ethic commitee of Shanghai Pulmonary Hospital, Tongji University<br>No. 507 Zhengmin Road, Yangpu District<br>shanghai, 200433<br>CHINA
1102	Yong Wang		Lina Jia<br>Zhihua Shi	Beijing Shijitan Hospital<br>Respiration Department<br>No.10,Yangfangdian Tieyeyuan Road, Haidian District<br>Beijing, 100038<br>CHINA	Ethic commitee of Beijing Shijitan Hospital<br>No.10 Yangfangdian Tieyiyuan Road, Haidian District<br>Beijing, 100038<br>CHINA

090177e181b842ab\Approved\Approved On: 17-Mar-2011 06:18

090177e181b842ab\Approved\Approved On: 17-Mar-2011 06:18

Denmark

Coordinating Investigators:
<None Entered>

Center	Principal Investigator	Co-Investigator(s)	Sub-Investigator(s)	Address(es)	Institutional Review Board or Ethics Committee Address(es)
1001 *	Dr. Jorn Carlsen			Hjertecentret Afs 2142<br>H S Rigshospitalet<br>Blegdamsvej 9<br>Kobenhavn, 2100<br>DENMARK	De Videnskabsetiske Komitéer for<br>Region Hovedstaden<br>Regionsgaarden<br>Kongens Vaenge 2<br>Hilleroed, 3400<br>DENMARK

090177e181b842ab\Approved\Approved On: 17-Mar-2011 06:18

Greece

Coordinating Investigators:
<None Entered>

Center	Principal Investigator	Co-Investigator(s)	Sub-Investigator(s)	Address(es)	Institutional Review Board or Ethics Committee Address(es)
1060	Assoc. Prof. Stylianos Orfanos		Dr. Anastasia Anthi<br>Assoc. Prof. Apostolos Armaganidis<br>Dr. Effrosyni Dimitriadou<br>John P. Lekakis<br>Dr. Panagiotis Lymperopoulos<br>Dr. Effrosyni Manali<br>Dr. Fotis Panou<br>Dr. Loukianos Rallidis<br>Dr. Helen Triantafyllidi<br>Dr. Iraklis Tsagkaris	Attikon Hospital<br>Second Critical Care Department,<br>University of Athens<br>1, Rimini St.<br>Haidari, Athens 12462<br>GREECE	National Ethics Committee<br>284 Mesogion Avenue<br>Athens, 15562<br>GREECE
1061 *	Dr. Athanassios Manginas		Prof. Dennis Cokkinos<br>Dr. Panagiotis Karyofyllis<br>Dr. Sofia Thomopoulou	Onassis Cardiac Surgery Center<br>1st Cardiology Clinic<br>356 Sygrou Avenue<br>Kallithea, Athens 176 74<br>GREECE	National Ethics Committee<br>284 Mesogion Av.<br>ATHENS, 15562<br>GREECE
1062 *	Prof. Lazaros Sichletidis		Dr. Aikaterini Manika<br>Dr. Dionisis Spyratos	Papanikolaou Hospital<br>University Pneumology Clinic<br>Asvestohori-Eksohi<br>Thessaloniki, 57010<br>GREECE	National Ethics Committee<br>284 Mesogion Avenue<br>Athens, 15562<br>GREECE

* Did not randomize subjects

India

Coordinating Investigators:
<None Entered>

Center	Principal Investigator	Co-Investigator(s)	Sub-Investigator(s)	Address(es)	Institutional Review Board or Ethics Committee Address(es)
1024	Dr. Kutumba Srinivasa Sastry Bhagavatula		Ms. Kundana Gogulamudi<br>Ms. Deepthi Kodali<br>Dr. Srinivas A. Kumar<br>Dr. Nirmal Kumar<br>Ms. Sandhya Rani<br>Ms. Sreelekha Sudanagunta<br>Dr. Suman Vyas Yandamury	Care Hospital, The Institute of Medical Sciences<br>Exhibition Road<br>Nampally<br>Hyderabad, Andhra Pradesh 500 001<br>INDIA	Institutional Ethical Committee, CARE Foundation<br>CARE Hospital<br>Road No. 1<br>Banjara Hills<br>Hyderabad, Andhra Pradesh 500 034<br>INDIA

090177e181b842ab\Approved\Approved On: 17-Mar-2011 06:18

Center	Principal Investigator	Co-Investigator(s)	Sub-Investigator(s)	Address(es)	Institutional Review Board or Ethics Committee Address(es)
1055	Dr. Kiron Varghese<br>Dr. Shamanna Seshadhri Iyengar (Previous PI)		Dr. Srilakshmi Mandayam Adhyapak<br>Dr. Mark Christopher Arokiraj<br>Dr. Yeriswamy Mogalahally Channabasappa<br>Dr. Balachandra Sagar Ganapathi<br>Dr. Cherian George<br>Dr. Santosh Mysore Jayadev<br>Ms. Beena Kuttarappally Sebastian<br>Dr. Thirumalesha Rattihalli Lakshmana Setty<br>Dr. Prashanth Devappa Naik<br>Ms. Preethi Muniyappa Narayanappa<br>Ms. Mamatha Kadathanamale Narayanappa<br>Dr. Poonam Nayak<br>Dr. Chandrakanth Bharamagouda Patil<br>Dr. Sandeep Patil<br>Dr. Amar R. Prabhudesai<br>Dr. Blessan Varghese Pulluvilayil<br>Dr. Shilpa Ramesh<br>Dr. Srikanth Secunlapuram Durga Prasad<br>Dr. Sandesh Seetharamappa<br>Dr. Gurappa Gojanur Shetty<br>Ms. Leena Singh<br>Ms. Arpita Taran<br>Dr. Chaya Devarahalli Vasudev<br>Ms. Ashita Yaji<br>Dr. Jayakeerthi Yoganarasimha	St. John's Medical College Hospital<br>Department of Cardiology, III Floor,<br>Sarjapur Road<br>Koramangala<br>Bangalore, Karnataka 560 034<br>INDIA	Institutional Ethical Review Board<br>St. John's Medical College and Hospital<br>Sarjapur Road<br>Bangalore, Karnataka 560 034<br>INDIA

090177e181b842ab\Approved\Approved On: 17-Mar-2011 06:18

090177e181b842ab\Approved\Approved On: 17-Mar-2011 06:18

Center	Principal Investigator	Co-Investigator(s)	Sub-Investigator(s)	Address(es)	Institutional Review Board or Ethics Committee Address(es)
1057	Dr. Parthiv M. Mehta		Dr. Roocha Majmundar Mehta<br>Mr. Poonam Nadia<br>Dr. Jigar J. Parikh<br>Dr. Jayesh R. Rawal	Mehta Hospital & Cardiopulmonary Care Center<br>2nd floor, Siddhachal Complex<br>Nr. Doordarshan Kendra<br>Drive-in-Road<br>Ahmedabad, Gujarat 380 054<br>INDIA	Safe Search Independent Ethics Committee<br>Siddhachal Complex, Nr. Doordarshan Kendra<br>Thaltej<br>Ahmedabad, Gujarat 380 054<br>INDIA
1058	Dr. Deepak Talwar		Mr. Mir Shad Ali<br>Dr. Ramesh Arora<br>Dr. Sharad Joshi<br>Dr. Sandeepan Kumar<br>Dr. Vipul Misra<br>Dr. Deepak Pabreja	Metro Multispeciality Hospital<br>L-94, Sector 11<br>Noida, Uttar Pradesh 201301<br>INDIA	Metro Ethics Review Board<br>Metro Hospitals & Heart Institutes<br>X-1, Sector 12<br>Noida, Uttar Pradesh 201 301<br>INDIA
1059	Dr. Darshan Banker		Dr. Nirav Bhalani<br>Dr. Sonia Dalal<br>Mr. Farhain Syed Masoovi<br>Ms. Jesal S. Nanavati<br>Dr. Manoj M. Parmar<br>Dr. Dharmesh Patel<br>Mr. Kamlesh Shah<br>Ms. Toral Sheth<br>Dr. Manoj Subramaniam<br>Dr. Narendrasingh Tanwar	Bankers Heart Institute<br>Near Tagorenagar<br>Opp. Suryakiran Complex<br>Old Padra Road<br>Vadodara, Gujarat 390 015<br>INDIA	Bankers Ethics Committee<br>Bankers Heart Institute<br>Near Tagorenagar<br>Opp. Suryakiran Complex<br>Old Padra Road<br>Vadodara, Gujarat 390 015<br>INDIA

090177e181b842ab\Approved\Approved On: 17-Mar-2011 06:18

Italy

Coordinating Investigators:
<None Entered>

Center	Principal Investigator	Co-Investigator(s)	Sub-Investigator(s)	Address(es)	Institutional Review Board or Ethics Committee Address(es)
1040	Prof. Carmine Dario Vizza		Dr. Roberto Badagliacca<br>Prof. Francesco Fedele<br>Dr. Roberto Poscia	Unita' di Ipertensione Polmonare, Dipartimento di Scienze Respiratorie e Cardiovascolari Universita' degli Studi di Roma La Sapienza<br>Viale del Policlinico, 155<br>Roma, 00161<br>ITALY	Comitato Etico Azienda Policlinico Umberto I<br>Viale del Policlinico, 155<br>Roma, 00155<br>ITALY
1043	Dr. Carlo Campana (Previous PI)<br>Dr. Stefano Ghio		Giulia Magrini<br>Laura Scelsi<br>Dr. Paolo Vicinelli	IRCCS Policlinico San Matteo<br>Dipartimento di Cardiologia<br>Piazzale Golgi, 2<br>Pavia, 27100<br>ITALY	Comitato di Bioetica della Fondazione IRCCS Policlinico S. Matteo di Pavia<br>Viale Golgi, 19<br>Pavia, 27100<br>ITALY
1067 *	Dr. Giuseppe Marazzi		Dr Elena Cerquetani<br>Francesco Pelliccia	IRCCS San Raffaele Pisana<br>Dipartimento di Cardiologia<br>Via della Pisana, 235<br>Roma, 00163<br>ITALY	Comitato Etico IRCCS San Raffele Pisana<br>Via di Val Cannuta, 247<br>Roma, 00166<br>ITALY
1089 *	Dr. Maria Paola Cicini<br>Dr. Pietro Tanzi (Previous PI)		Maria Giuseppina Mastrantuono<br>Dr. Bruno Polletta<br>Dr. Pietro Tanzi	Azienda Ospedaliera San Camillo di Roma<br>Dipartimento Cardio Scienze Cardiologia III<br>Circonvallazione Gianicolense, 87<br>Roma, 00152<br>ITALY	Comitato Etico dell'Azienda Ospedaliera San Camillo Forlanini di Roma<br>Circonvallazione Gianicolense, 87<br>Roma, 00152<br>ITALY

* Did not randomize subjects

Center	Principal Investigator	Co-Investigator(s)	Sub-Investigator(s)	Address(es)	Institutional Review Board or Ethics Committee Address(es)
1090 *	Dr. Patrizio Vitulo		Dr. Lorenza Conti<br>Dr. Simona Soresi	Servizio di Pneumologia, Istituto Mediterraneo per i Trapianti e Terapie ad Alta Specializzazione<br>Via Ernesto Tricomi, 1<br>Palermo, 90147<br>ITALY	Comitato Etico Istituto Mediterraneo per Trapianti e Terapie Alta Specializzazione ISMETT di Palermo<br>Via Ernesto Tricomi, 1<br>Palermo, 90127<br>ITALY

090177e181b842ab\Approved\Approved On: 17-Mar-2011 06:18

* Did not randomize subjects

Latvia

Coordinating Investigators:
<None Entered>

Center	Principal Investigator	Co-Investigator(s)	Sub-Investigator(s)	Address(es)	Institutional Review Board or Ethics Committee Address(es)
1081	Prof. Andrejs Erglis		Dr. Ainars Rudzitis<br>Dr. Andris Skride	P. Stradins Clinical University Hospital / Latvian Centre of Cardilogy<br>Pilsonu Street 13<br>Riga, LV - 1002<br>LATVIA	Ethics Committee For Clinical Research of Medicines and Pharmaceutical Products<br>Paula Stradina Clinical University Hospital Development Foundation<br>Pilsonu Street 13<br>Riga, LV 1002<br>LATVIA

090177e181b842ab\Approved\Approved On: 17-Mar-2011 06:18

Malaysia

Coordinating Investigators:
<None Entered>

Center	Principal Investigator	Co-Investigator(s)	Sub-Investigator(s)	Address(es)	Institutional Review Board or Ethics Committee Address(es)
1096	Dato' Dr Amin Ariff Nuruddin		Datuk Dr Aizai Azan Abdul Rahim<br>Dr Geetha Kandavello	National Heart Institute<br>145, Jalan Tun Razak<br>Kuala Lumpur, 50400<br>MALAYSIA	Institut Jantung Negara Ethics Committee<br>National Heart Institute<br>145,<br>Jalan Tun Razak<br>Kuala Lumpur, Kuala Lumpur 50400<br>MALAYSIA

090177e181b842ab\Approved\Approved On: 17-Mar-2011 06:18

Netherlands

Coordinating Investigators:
<None Entered>

Center	Principal Investigator	Co-Investigator(s)	Sub-Investigator(s)	Address(es)	Institutional Review Board or Ethics Committee Address(es)
1018	Dr. Anko Boonstra		Dr. Antonie Vonk Noordergraaf	VU Medisch Centrum / afdeling Longziekten<br>De Boelelaan 1117<br>Amsterdam, 1081 HV<br>NETHERLANDS	Vrije Universiteit Medisch Centrum<br>Medisch Ethische Toetsingscommissie<br>6 Z 192<br>De Boelelaan 1117<br>Amsterdam, 1081 HV<br>NETHERLANDS
1065	Dr. Leon M. van den Toorn		Dr. Marlies Wijsenbeek-Lourens	Erasmus MC<br>Kamer SV-017<br>Dr. Molenwaterplein 40<br>Rotterdam, 3015 GE<br>NETHERLANDS	Vrije Universiteit Medisch Centrum<br>Medisch Ethische Toetsingscommissie<br>6 Z 192<br>De Boelelaan 1117<br>Amsterdam, 1081 HV<br>NETHERLANDS

090177e181b842ab\Approved\Approved On: 17-Mar-2011 06:18

090177e181b842ab\Approved\Approved On: 17-Mar-2011 06:18

Panama

Coordinating Investigators:
<None Entered>

Center	Principal Investigator	Co-Investigator(s)	Sub-Investigator(s)	Address(es)	Institutional Review Board or Ethics Committee Address(es)
1100 *	Daniel Pichel		Rafael Restrepo	Cardiologos Asociados de Panama<br>Consultorios Medicos Paitilla<br>Calle 53, Avenida Balboa<br>Panama City, Panama<br>PANAMA	Instituto Conmemorativo Gorgas<br>Comite Nacional de Bioetica en Investigacion<br>AV. Justo Arosemena<br>calle 35 y 36<br>Panama City, Panama 6991<br>PANAMA

* Did not randomize subjects

Philippines

Coordinating Investigators:
<None Entered>

Center	Principal Investigator	Co-Investigator(s)	Sub-Investigator(s)	Address(es)	Institutional Review Board or Ethics Committee Address(es)
1082	Dr. Ma. Encarnita B. Limpin		Dr. Erma D. Garcia-Lazaro	Philippine Heart Center<br>Pulmonary Rehabilitation Section<br>8th Floor Medical Arts Building<br>East Avenue<br>Quezon City, 1100<br>PHILIPPINES	Philippine Heart Center-Institutional Review Board<br>Philippine Heart Center<br>8th Floor Medical Arts Building<br>East Avenue<br>Quezon City, 1100<br>PHILIPPINES
1087	Dr. Jubert P. Benedicto		Dr Norman L. Maghuyop	Philippine General Hospital<br>Central Adult and Pediatric Intensive Care Unit<br>2nd Floor Central Block Building<br>Pedro Gil Street, Ermita Taft Avenue<br>Manila City, 1000<br>PHILIPPINES	Research Implementation and Development Office<br>Philippine General Hospital- UP College of Medicine<br>547 Floor Paz Mendoza Building<br>Pedro Gil Street, Ermita<br>Manila, 1000<br>PHILIPPINES

090177e181b842ab\Approved\Approved On: 17-Mar-2011 06:18

090177e181b842ab\Approved\Approved On: 17-Mar-2011 06:18

Poland

Coordinating Investigators:
<None Entered>

Center	Principal Investigator	Co-Investigator(s)	Sub-Investigator(s)	Address(es)	Institutional Review Board or Ethics Committee Address(es)
1036 *	Dr. Marcin Kurzyna		Dr. Anna Fijalkowska<br>Dr. Michal Florczyk<br>Dr. Maria Wieteska	Instytut Gruzlicy i Chorob Pluc<br>Klinika Chorob Wewnetrznych<br>Klatki Piersiowej<br>ul. Plocka 26<br>Warszawa, 01-138<br>POLAND	Komisja ds. Etyki Badan Naukowych<br>Przy Instytucie Gruzlicy i Chorob<br>Pluc<br>ul. Plocka 26<br>Warszawa, 01-138<br>POLAND
1037	Prof. Piotr Podolec		Dr. Magdalena Kaznica<br>Dr. Magdalena Nowacka<br>Dr. Wieslawa Tracz<br>Dr. Piotr Wilkolek<br>Magdalena Wojtowicz-<br>Kaczmarczyk	Krakowski Szpital<br>Specjalistyczny Im. Jana Pawla II<br>w Krakowie<br>Oddzial Kliniczny Chorob Serca<br>i Naczyn<br>Ul. Pradnicka 80<br>Krakow, 31-202<br>POLAND	Komisja ds. Etyki Badan Naukowych<br>Przy Instytucie Gruzlicy i Chorob<br>Pluc<br>ul. Plocka 26<br>Warszawa, 01-138<br>POLAND
1071 *	Prof. Lech Polonski		Dr. Bartosz Hudzik<br>Dr. Anna Kazik<br>Dr. Mateusz Ostrega<br>Dr. Jacek Piegza	Samodzielny Publiczny Zaklad<br>Opieki Zdrowotnej, Slaskie<br>Centrum Chorob Serca<br>III Katedra i Oddzial Kliniczny<br>Kardiologii Slaskiego<br>Uniwersytetu Medycznego w<br>Katowicach<br>Ul. Szpitalna 2<br>Zabrze, 41-800<br>POLAND	Komisja ds. Etyki Badan Naukowych<br>Przy Instytucie Gruzlicy i Chorob<br>Pluc<br>ul. Plocka 26<br>Warszawa, 01-138<br>POLAND

* Did not randomize subjects

090177e181b842ab\Approved\Approved On: 17-Mar-2011 06:18

Romania

Coordinating Investigators:
<None Entered>

Center	Principal Investigator	Co-Investigator(s)	Sub-Investigator(s)	Address(es)	Institutional Review Board or Ethics Committee Address(es)
1097	Prof.Dr. Voicu Mircea Tudorache		Dr. Nicoleta Sorina Bertici<br>Dr. Ionela Iovan<br>Dr. Dorin Vancea	Spitalul Clinic de Boli Infectioase si Pneumoftiziologie Dr. Victor Babes<br>Str. Gheorghe Adam nr. 13<br>Timisoara, Timis<br>ROMANIA	Academia de Stiinte Medicale,Comisia Nationala de Etica pentru Studiul Clinic al Medicamentului<br>Str. Av. Sanatescu nr. 48,<br>Sector 1,<br>Bucuresti, 011478<br>ROMANIA
1099 *	Prof.Dr. Traian Mihaescu		Raluca Mihaela Bercea<br>Dr. Anda Tesloianu	Spitalul Clinic de Pneumoftiziologie<br>Str.Iosif Cihac, nr 30<br>Iasi, Iasi 700115<br>ROMANIA	Academia de Stiinte Medicale,Comisia Nationala de Etica pentru Studiul Clinic al Medicamentului<br>Str. Av. Sanatescu nr. 48,<br>Sector 1,<br>Bucuresti, 011478<br>ROMANIA

* Did not randomize subjects

090177e181b842ab\Approved\Approved On: 17-Mar-2011 06:18

Russian Federation

Coordinating Investigators:
<None Entered>

Center	Principal Investigator	Co-Investigator(s)	Sub-Investigator(s)	Address(es)	Institutional Review Board or Ethics Committee Address(es)
1045 *	Dr. Evgene N. Semernin		Dr. Alexandra Y. Gudkova<br>Dr. Svetlana Kozlova<br>Dr. Raisa Polozova	Almazov Federal Heart, Blood and Endocrinology Centre<br>2, Akkuratova str.<br>Saint-Petersburg, 197341<br>RUSSIAN FEDERATION<br><br>Almazov Federal Heart, Blood and Endocrinology Centre<br>Clinical-Rehabilitation complex<br>15, Parkhomenko str.<br>Saint-Petersburg, 194156<br>RUSSIAN FEDERATION<br><br>Almazov Federal Heart, Blood and Endocrinology Centre<br>Cardio Surgery unit<br>1, Severniy prospect.<br>Saint-Petersburg, 194354<br>RUSSIAN FEDERATION<br><br>S-Petersburg State Medical University named after academic I.P. Pavlov<br>Lva Tolstogo str. 6/8<br>Saint-Petersburg, 197022<br>RUSSIAN FEDERATION	Ethics Committee<br>Saint-Petersburg State Medical University named after I.P. Pavlov of Roszdrav<br>Roentgen str., 10<br>Saint-Petersburg, 197101<br>RUSSIAN FEDERATION<br><br>Ethics Committee within the Federal Authority for Healthcare and Social Development Regulation<br>8, str. 2, Petrovskij bulvar<br>Moscow, 127051<br>RUSSIAN FEDERATION

090177e181b842ab\Approved\Approved On: 17-Mar-2011 06:18

Center	Principal Investigator	Co-Investigator(s)	Sub-Investigator(s)	Address(es)	Institutional Review Board or Ethics Committee Address(es)
1046	Prof. Sergey V. Gorbachevsky		Dr. Victoriya Victorovna Sheverdina<br>Dr. Karina Y. Tatevosyan	Scientific Center of Cardiovascular surgery n.a. A.N.Bakoulev RAMS<br>Rublevskoye shosse, 135<br>Moscow, 121552<br>RUSSIAN FEDERATION<br><br>Scientific Center of Cardiovascular surgery n.a. A.N.Bakoulev RAMS<br>Leninskiy pr. 8<br>Moscow, 117931<br>RUSSIAN FEDERATION	Ethics Committee within the Federal Authority for Healthcare and Social Development Regulation<br>8, str. 2, Petrovskij bulvar<br>Moscow, 127051<br>RUSSIAN FEDERATION
1083 *	Prof. Vitaly Berezin		Nataliya A. Egorova<br>Natalia G. Kuleshova<br>Viktoria V. Vereshchagina<br>Valery S. Verkhovskiy	Saint Petersburg's City Hospital #26, Department of Cardiology #2.<br>2. Kostyushko str.<br>Saint Petersburg, 196247<br>RUSSIAN FEDERATION	Ethics Committee of Saint Petersburg's City Hospital #26<br>Ulitsa Kostushko, 2<br>Saint Petersburg, 196247<br>RUSSIAN FEDERATION<br><br>Ethics Committee within the Federal Authority for Healthcare and Social Development Regulation<br>8, str. 2, Petrovskij bulvar<br>Moscow, 127051<br>RUSSIAN FEDERATION

* Did not randomize subjects

090177e181b842ab\Approved\Approved On: 17-Mar-2011 06:18

Thailand

Coordinating Investigators:
<None Entered>

Center	Principal Investigator	Co-Investigator(s)	Sub-Investigator(s)	Address(es)	Institutional Review Board or Ethics Committee Address(es)
1093	Prof. Ratanavadee Nanagara		Assist.Prof Chingching Foocharoen<br>Assist.Prof. Ajanee Mahakkanukrauh<br>Assoc.Prof Siraphop Suwannaroj	Division of Rheumatology Allergy and Immunology, Department of Medicine, Faculty of Medicine<br>Khon Kaen University<br>123 Mitraparb Road,<br>Amphoe Mueang, Khon Kaen 40002<br>THAILAND	The Khon Kaen University Ethics Committee for Human Research<br>Faculty of Medicine, Khon Kaen University<br>Dean Office 6 floor<br>123 Mitraphap Road<br>Khon Kaen, 40200<br>THAILAND
1094	Dr. Taworn Suithichaiyakul			Department of Medicine, Chulalongkorn University<br>1873 Rama 4 Road, Pathumwan<br>Bangkok, Thailand 10330<br>THAILAND	Institutional Review Board, Faculty of Medicine, Chulalongkorn University<br>1873 Rama IV Road<br>Pathumwan<br>Bangkok, Bangkok 10330<br>THAILAND

090177e181b842ab\Approved\Approved On: 17-Mar-2011 06:18

United Kingdom

Coordinating Investigators:
<None Entered>

Center	Principal Investigator	Co-Investigator(s)	Sub-Investigator(s)	Address(es)	Institutional Review Board or Ethics Committee Address(es)
1085	Professor Paul A. Corris		Dr. Andrew J. Fisher<br>Dr. James L. Lordan<br>Dr. Guy Andrew Macgowan	Room 224A Sir William Leech Centre<br>Freeman Hospital<br>Freeman Road<br>Newcastle Upon Tyne, Tyne and Wear NE7 7DN<br>UNITED KINGDOM	South West 2 REC<br>Royal Devon & Exeter Hospital (Heavitree)<br>Research Ethics Service<br>Gladstone Road<br>Exeter, Devon EX1 2ED<br>UNITED KINGDOM
1088	Dr. Leisa Freeman		Dr. Alice L. Wood	Norfolk and Norwich University Hospital<br>Clinical Research and Trials Unit<br>Cardiology Department<br>Level 3, East Block<br>Colney Lane<br>Norwich, Norfolk NR4 7UY<br>UNITED KINGDOM	South West 2 REC<br>Royal Devon & Exeter Hospital (Heavitree)<br>Research Ethics Service<br>Gladstone Road<br>Exeter, Devon EX1 2ED<br>UNITED KINGDOM

090177e181b842ab\Approved\Approved On: 17-Mar-2011 06:18

United States

Coordinating Investigators:
<None Entered>

Center	Principal Investigator	Co-Investigator(s)	Sub-Investigator(s)	Address(es)	Institutional Review Board or Ethics Committee Address(es)
1013 *	Dr. Paul Bennett deBoisblanc		Dr. Suma D. Jain<br>Dr. Hamang M. Patel<br>Dr. Warren R. Summer<br>Dr. Hector Osvaldo Ventura	LSUHSC<br>Room 6B1<br>1900 Gravier Street<br>New Orleans, LA 70112<br>UNITED STATES<br><br>Medical Center of Louisiana - University Hospital - CTRC<br>2021 Gravier Street<br>New Orleans, LA 70112<br>UNITED STATES	LSU Health Sciences Center<br>Institutional Review Board<br>433 Bolivar Street<br>New Orleans, LA 70112<br>UNITED STATES
1016	Dr. Robert Michael Aris		Dr. Hubert James Ford	The University of North Carolina at Chapel Hill<br>4th Floor BioInformatics Building<br>CB# 7020<br>130 Mason Farm Road<br>Chapel Hill, NC 27599<br>UNITED STATES	Office of Human Research Ethics (OHRE)<br>BioMedical Institute Review Board<br>CB #7097<br>Medical School Building 52<br>Chapel Hill, NC 27599-7097<br>UNITED STATES
1017 *	Dr. Roblee Peter Allen		Dr. Timothy Eugene Albertson<br>Dr. Mark V. Avdalovic	University of California Davis Medical Center<br>Suite 3400<br>4150 V Street<br>Sacramento, CA 95817<br>UNITED STATES	University of California Davis<br>Institutional Review Board<br>University of California Medical Center - Crisp Building<br>2921 Stockton Boulevard<br>Sacramento, CA 95817<br>UNITED STATES

* Did not randomize subjects

Center	Principal Investigator	Co-Investigator(s)	Sub-Investigator(s)	Address(es)	Institutional Review Board or Ethics Committee Address(es)
1021	Dr. Zeenat Safdar		Dr. Wayne J. Franklin<br>Dr. Adaani Ethel Frost	Baylor College of Medicine<br>Pulmonary Section<br>Suite 1225<br>6620 Main Street<br>Houston, TX 77030<br>UNITED STATES<br><br>St. Luke's Episcopal Hospital<br>6720 Bertner Avenue<br>Houston, TX 77030<br>UNITED STATES	BCM Office of Research<br>Institutional Review Board for Human Subjects<br>Room 600D<br>One Baylor Plaza<br>Houston, TX 77030<br>UNITED STATES
1030	Dr. Jeremy P Feldman		Dr. Gregory Scott Ahearn<br>Dr. Shawn E. Wright	Arizona Pulmonary Specialists, LTD<br>Suite 950<br>500 West Thomas Road<br>Phoenix, AZ 85013<br>UNITED STATES	Western Institutional Review Board, Inc.<br>3535 Seventh Avenue, SouthWest<br>Olympia, WA 98502<br>UNITED STATES
1031	Dr. Boaz A. Markewitz		Dr. Nathan D. Hatton<br>Dr. Ernst-Gilbert Schreiber	University of Utah Sciences Center<br>50 North Medical Drive<br>Salt Lake City, UT 84132<br>UNITED STATES	University of Utah IRB<br>512 RAB<br>75 South 2000 East<br>Salt Lake City, UT 84112<br>UNITED STATES

090177e181b842ab\Approved\Approved On: 17-Mar-2011 06:18

* Did not randomize subjects

Center	Principal Investigator	Co-Investigator(s)	Sub-Investigator(s)	Address(es)	Institutional Review Board or Ethics Committee Address(es)
1032	Dr. Jamie Cooper Hey		Dr. Glenn Matthew Giessel<br>Dr. Shaival J. Kapadia<br>Dr. Ritsu Kuno<br>Dr. James A.L. Mathers<br>Dr. Calvin Mark Newton<br>Dr. Michael B. Polsky<br>Dr. Cullen B. Rivers<br>Dr. Pawanjit S. Sarna<br>Dr. Rodney H. Smith<br>Dr. Peter F. Torrisi	Cardiovascular Associates of Virginia<br>Imaging Only<br>Suite 200<br>7001 Forest Avenue<br>Richmond, VA 23230<br>UNITED STATES<br><br>CJW Chippenham Medical Center<br>7101 Jahnke Road<br>Richmond, VA 23225<br>UNITED STATES<br><br>Pulmonary Associates of Richmond, Inc.<br>Suite 201<br>1000 Boulders Parkway<br>Richmond, VA 23225<br>UNITED STATES<br><br>Virginia Cardiovascular Specialists<br>Suite 100<br>7401 Beaufont Springs Drive<br>Richmond, VA 23225<br>UNITED STATES	Chippenham & Johnston-Willis Medical Center<br>Institutional Review Board<br>7101 Jahnke Road<br>Richmond, VA 23225<br>UNITED STATES
1048 *	Dr. Nathaniel Marchetti (Previous PI)<br>Dr. Sheila Elizabeth Weaver		Dr. Francis Chan Cordova<br>Dr. Gerard Joseph Criner<br>Dr. Nathaniel Marchetti<br>Dr. Brian O'Murchu	Temple Lung Center<br>Parkinson Pavilion, 7th Floor<br>3401 North Broad Street<br>Philadelphia, PA 19140<br>UNITED STATES<br><br>Temple University Hospital<br>3401 North Broad Street<br>Philadelphia, PA 19140<br>UNITED STATES	Western Institutional Review Board<br>3535 Seventh Avenue Southwest<br>Olympia, WA 98502<br>UNITED STATES

* Did not randomize subjects

090177e181b842ab\Approved\Approved On: 17-Mar-2011 06:18

090177e181b842ab\Approved\Approved On: 17-Mar-2011 06:18

Center	Principal Investigator	Co-Investigator(s)	Sub-Investigator(s)	Address(es)	Institutional Review Board or Ethics Committee Address(es)
1050 *	Dr. Adam Lawrence Greene		Dr. David C. Mares	Saint John's Research Institute<br>Suite 110<br>2020 Meridian Street<br>Anderson, IN 46016<br>UNITED STATES	St. Vincent Institutional Review Board<br>Suite 208<br>8402 Harcourt Road<br>Indianapolis, IN 46260<br>UNITED STATES
1052 *	Dr. Daniel Stevens Fuleihan		Dr. Ziad A. El-Khally<br>Robert D. Kirk	New York Heart Center<br>Suite 300<br>1000 East Genesee Street<br>Syracuse, NY 13210<br>UNITED STATES	Saint Joseph's Hospital<br>Human Research Committee<br>301 Prospect Avenue<br>Syracuse, NY 13203<br>UNITED STATES
1053	Dr. Anantharam V. Kalya		Kristen L. Combes<br>Nadine C. Henning<br>Dr. Thomas P. Schleeter<br>Dr. Mary Norine Walsh	The Care Group, LLC<br>8333 Naab Road<br>Indianapolis, IN 46260<br>UNITED STATES	Western Institutional Review Board, Inc.<br>3535 Seventh Avenue SW<br>Olympia, WA 98502-5010<br>UNITED STATES
1054 *	Dr. Mohammad Waseem Farra		Dr. Harvey W. Organek	Consultants in Sleep and Pulmonary Medicine<br>28200 Franklin Road<br>Southfield, MI 48034<br>UNITED STATES<br><br>United Physicians Research Insititute<br>Suite 2800<br>30800 Telegraph Road<br>Bingham, MI 48025<br>UNITED STATES	United Physicians Research IRB<br>Suite 2800<br>30800 Telegraph Road<br>Bingham Farms, MI 48025<br>UNITED STATES

* Did not randomize subjects

090177e181b842ab\Approved\Approved On: 17-Mar-2011 06:18

Center	Principal Investigator	Co-Investigator(s)	Sub-Investigator(s)	Address(es)	Institutional Review Board or Ethics Committee Address(es)
1063	Dr. Jeffrey Evan Michaelson		Dr. Susanti R. Ie<br>Dr. Rana Rab-Hasan<br>Dr. Steven Michael Rosenthal<br>Dr. Chuansheng Jonathon Wu	Atlanta Institute for Medical Research, Inc.<br>Suite 220<br>495 Winn Way<br>Decatur, GA 30030<br>UNITED STATES	Western Institutional Review Board<br>3535 Seventh Avenue Southwest<br>Olympia, WA 98502<br>UNITED STATES
1064	Dr. James R. Gossage		Dr. Amy Renee Blanchard	Medical College of Georgia<br>BBR 5513<br>1120 15th Street<br>Augusta, GA 30912<br>UNITED STATES	Human Assurance Committee<br>Medical College of Georgia<br>CJ-2103<br>1120 15th Street<br>Augusta, GA 30912<br>UNITED STATES
1066 *	Dr. David H. Silber		Dr. John Phillip Boehmer<br>Patricia M. Frey<br>Laurie S. Huddy	Penn States Milton S. Hershey Medical Center<br>Heart and Vascular Insititute, Mail Code H047<br>500 University Drive<br>Hershey, PA 17033<br>UNITED STATES	Penn State Milton S. Hershey Medical Center<br>Human Subjects Protection Office/Institutional Review Board<br>Mail Code A115<br>600 Centerview Drive<br>Hershey, PA 17033<br>UNITED STATES
1068 *	Dr. Lev Mark Khitin		Dr. Yan Katsnelson	Chicago Heart Institute<br>Suite 201<br>800 Biesterfield Road<br>Elk Grove Village, IL 60007<br>UNITED STATES	Western Institutional Review Board<br>3535 Seventh Avenue Southwest<br>Olympia, WA 98502<br>UNITED STATES

* Did not randomize subjects

Center	Principal Investigator	Co-Investigator(s)	Sub-Investigator(s)	Address(es)	Institutional Review Board or Ethics Committee Address(es)
1077	Dr. Kevin Cronin Sharkey		Dr. John Edward Alexander<br>Dr. Edward Beman McMillan	Mid Carolina Cardiology<br>Suite 501 and Suite 605<br>1718 East Fourth Street<br>Charlotte, NC 28204<br>UNITED STATES<br><br>Presbyterian Hospital<br>200 Hawthorne Lane<br>Charlotte, NC 28204<br>UNITED STATES	Presbyterian Hospital IRB<br>PO Box 33549<br>200 Hawthorne Lane<br>Charlotte, NC 28233-3549<br>UNITED STATES
1078 *	Dr. Ronald T. Uszenski		Dr. Jonathan Eliot Este Fisher<br>Dr. George Vernon Irons Jr.<br>Dr. James David Kay<br>Dr. Kenneth Durham Weeks Jr.	Mid Carolina Cardiology<br>Suite 201<br>10030 Gilead Road<br>Huntersville, NC 28078<br>UNITED STATES<br><br>Presbyterian Hospital Huntersville<br>10030 Gilead Road<br>Huntersville, NC 28078<br>UNITED STATES	Presbyterian Hospital IRB<br>PO Box 33549<br>200 Hawthorne Lane<br>Charlotte, NC 28233-3549<br>UNITED STATES

* Did not randomize subjects

090177e181b842ab\Approved\Approved On: 17-Mar-2011 06:18
