# Supplementary material for: Efficacy of 1, 5, and 20 mg oral sildenafil in the treatment of adults with pulmonary arterial hypertension: a randomized, double-blind study with open-label extension
Source: BMC Pulm Med. 2017 Feb 23;17:44. doi: 10.1186/s12890-017-0374-x (PMC5322647; doi:10.1186/s12890-017-0374-x)
Supplement: Additional file 2: — Supplemental Methods. Methods for LOCF, time to clinical worsening (TTCW), and Borg assessments. (DOCX 14 kb) [file 12890_2017_374_MOESM2_ESM.docx]

**Supplemental Material**

**Supplemental Methods**

The effect of sildenafil 20 mg on functional class at 12 and 24 weeks (LOCF) was compared with the 2 lower doses by using proportional odds logistic regression. Time to clinical worsening (TTCW) was to be assessed using a Cox proportional hazard model. BNP and TAPSE index were analyzed at weeks 12 and 24, using the same method as for the secondary endpoints.

Change from baseline to week 12 and week 24 in Borg score was assessed with a stratified Wilcoxon test (Van-Elteren). Borg assessments were valid for analysis only if 6MWD was performed on the same day. Correlations between change in 6MWD and BNP and pro-BNP data were made using Pearson correlation.

An interim analysis was specified when 50% of the planned patients had been randomized and completed 12 weeks of study (or had withdrawn). The study would be stopped for early evidence of a dose response. A Pocock-type alpha spending function was used to produce appropriate stopping boundaries to control the type 1 error.

The independent DMC convened on March 10, 2010, in response to the FDA release from the postapproval commitment. Primarily because sildenafil 20 mg TID had been shown to reduce TTCW and also acknowledging practical issues with recruiting that caused the study to be unlikely to meet enrollment, the DMC advised stopping the study. The DMC recommended ending recruitment with titration of all patients to the approved sildenafil dose (20 mg TID). No safety issues related to sildenafil treatment arose. The study was halted on June 3, 2010; all patients were provided treatment with sildenafil 20 mg TID if supported by their physician. A final analysis was performed on accrued data.
