# Supplementary material for: Efficacy of 1, 5, and 20 mg oral sildenafil in the treatment of adults with pulmonary arterial hypertension: a randomized, double-blind study with open-label extension
Source: BMC Pulm Med. 2017 Feb 23;17:44. doi: 10.1186/s12890-017-0374-x (PMC5322647; doi:10.1186/s12890-017-0374-x)
Supplement: Additional file 3: Figure S1. — Relationship of change from baseline in 6MWD and sildenafil average steady-state concentration. (DOCX 121 kb) [file 12890_2017_374_MOESM3_ESM.docx]

**Figure S1.** Relationship of change from baseline in 6MWD and sildenafil average steady-state concentration.


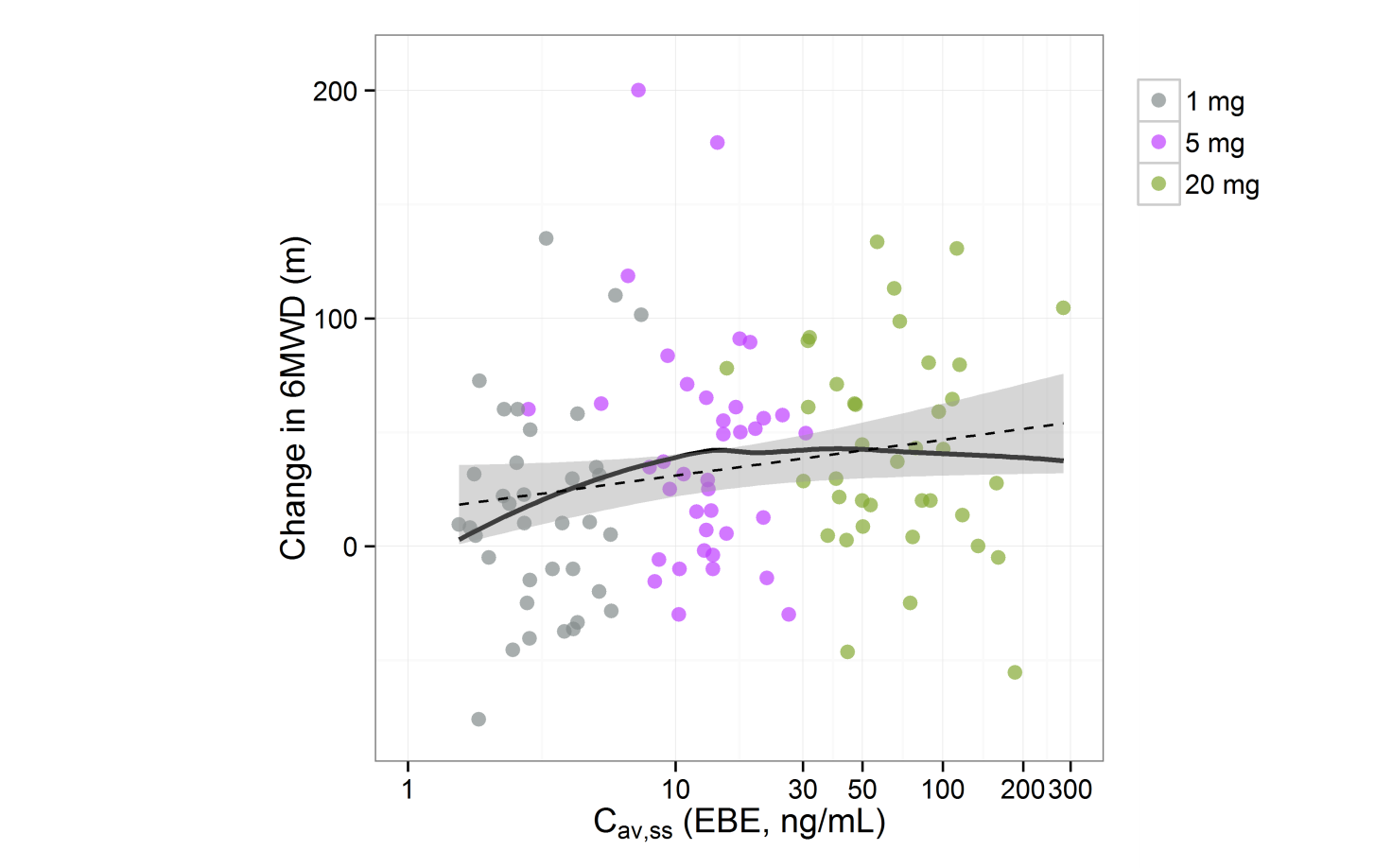


Symbols correspond to dose groups, the linear model and its 95% confidence interval is indicated by a straight dashed line and a shaded area, respectively, and the solid black line corresponds to a smoother curve.
