# Supplementary material for: Efficacy of 1, 5, and 20 mg oral sildenafil in the treatment of adults with pulmonary arterial hypertension: a randomized, double-blind study with open-label extension
Source: BMC Pulm Med. 2017 Feb 23;17:44. doi: 10.1186/s12890-017-0374-x (PMC5322647; doi:10.1186/s12890-017-0374-x)

**Figure S3.**  Plot of observed plasma sildenafil concentrations (open circles) vs time after sildenafil doses of 1 mg TID (bottom panels), 5 mg TID (middle panels), and 20 mg TID (top panels). Each column represents a subset of the measured concentrations: after the first dose (left column), after all subsequent doses (middle column), and after administration of cytochrome P450 (CYP) 3A4 inhibitors (right column). Median (solid line) and 90% prediction intervals (dashed lines) from simulations are overlaid. Tick marks on the horizontal time axis indicate concentration measures below the limit of quantification. TID=3 times daily. The concentration ranges aligned with the intent to produce differentiated pharmacologic effects.


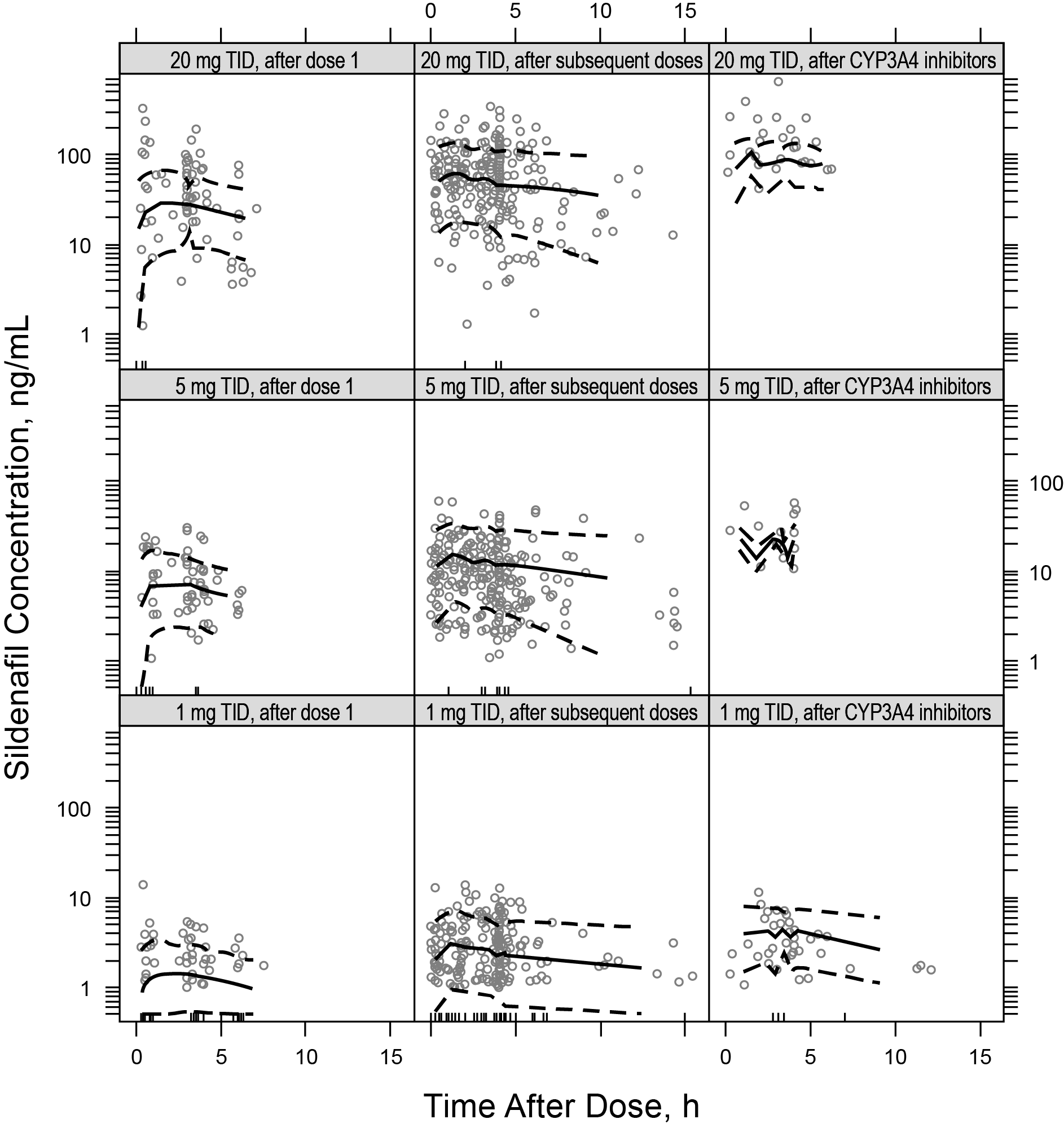

Supplement: Additional file 5: Figure S3. — Plot of observed plasma sildenafil concentrations (open circles) vs time after sildenafil doses of 1 mg TID (bottom panels), 5 mg TID (middle panels), and 20 mg TID (top panels). (DOCX 179 kb) [file 12890_2017_374_MOESM5_ESM.docx]
