# Supplementary material for: Efficacy of 1, 5, and 20 mg oral sildenafil in the treatment of adults with pulmonary arterial hypertension: a randomized, double-blind study with open-label extension
Source: BMC Pulm Med. 2017 Feb 23;17:44. doi: 10.1186/s12890-017-0374-x (PMC5322647; doi:10.1186/s12890-017-0374-x)
Supplement: Additional file 6: Table S1. — Results of a linear model, regressing 6MWD change from baseline against sildenafil average steady-state concentrations. (DOCX 14 kb) [file 12890_2017_374_MOESM6_ESM.docx]

**Table S1.** Results of a linear model, regressing 6MWD change from baseline against sildenafil average steady-state concentrations.

Call:

lm(formula = C6MWD ~ log10(CSS), data = dat2)

| **Residuals:** |  |  |  |  |
| --- | --- | --- | --- | --- |
| **Minimum** | **1Q** | **Median** | **3Q** | **Maximum** |
| -106.518 | -34.479 | -4.094 | 29.032 | 171.183 |
| **Coefficients:** |  |  |  |  |
|  | **Estimate** | **Standard Error** | **t value** | ***P*r(>\|t\|)** |
| (Intercept) | 15.238 | 10.054 | 1.516 | 0.1325 |
| log10(CSS) | 15.755 | 7.783 | 2.024 | 0.0453 * |

**P*=0.01

Residual standard error: 47.69 on 110 degrees of freedom

Multiple R-squared: 0.03592, Adjusted R-squared: 0.02715

F-statistic: 4.098 on 1 and 110 DF; *P*-value: 0.04535
