# Supplementary material for: Efficacy of 1, 5, and 20 mg oral sildenafil in the treatment of adults with pulmonary arterial hypertension: a randomized, double-blind study with open-label extension
Source: BMC Pulm Med. 2017 Feb 23;17:44. doi: 10.1186/s12890-017-0374-x (PMC5322647; doi:10.1186/s12890-017-0374-x)
Supplement: Additional file 7: Table S2. — Results of a linear model, regressing PVR change from baseline against sildenafil average steady state concentrations. (DOCX 14 kb) [file 12890_2017_374_MOESM7_ESM.docx]

**Table S2.** Results of a linear model, regressing PVR change from baseline against sildenafil average steady state concentrations.

Call:

lm(formula = CHG ~ log10(CSS), data = dat1)

| **Residuals:** |  |  |  |  |
| --- | --- | --- | --- | --- |
| **Minimum** | **1Q** | **Median** | **3Q** | **Maximum** |
| -1567.78 | -151.18 | 68.19 | 229.47 | 1337.76 |
| **Coefficients:** |  |  |  |  |
|  | **Estimate** | **Standard Error** | **tvalue** | ***P*r(>\|t\|)** |
| (Intercept) | -142.76 | 89.57 | -1.594 | 0.114 |
| log10(CSS) | -29.72 | 69.89 | -0.425 | 0.672 |

Residual standard error: 412.6 on 101 degrees of freedom

Multiple R-squared: 0.001788, Adjusted R-squared: -0.008096

F-statistic: 0.1809 on 1 and 101 DF; *P*=0.6715
